# Microbial and Enzymatic Degradation of Synthetic Plastics

**DOI:** 10.3389/fmicb.2020.580709

**Published:** 2020-11-26

**Authors:** Nisha Mohanan, Zahra Montazer, Parveen K. Sharma, David B. Levin

**Affiliations:** ^1^Department of Biosystems Engineering, University of Manitoba, Winnipeg, MB, Canada; ^2^Faculty of Food Engineering, The Educational Complex of Agriculture and Animal Science, Torbat-e-jam, Iran

**Keywords:** synthetic polymers, polyethylene, polyethylene terephthalate, polyurethane, polystyrene, polypropylene, polyvinyl chloride, cutinase

## Abstract

Synthetic plastics are pivotal in our current lifestyle and therefore, its accumulation is a major concern for environment and human health. Petroleum-derived (petro-)polymers such as polyethylene (PE), polyethylene terephthalate (PET), polyurethane (PU), polystyrene (PS), polypropylene (PP), and polyvinyl chloride (PVC) are extremely recalcitrant to natural biodegradation pathways. Some microorganisms with the ability to degrade petro-polymers under *in vitro* conditions have been isolated and characterized. In some cases, the enzymes expressed by these microbes have been cloned and sequenced. The rate of polymer biodegradation depends on several factors including chemical structures, molecular weights, and degrees of crystallinity. Polymers are large molecules having both regular crystals (crystalline region) and irregular groups (amorphous region), where the latter provides polymers with flexibility. Highly crystalline polymers like polyethylene (95%), are rigid with a low capacity to resist impacts. PET-based plastics possess a high degree of crystallinity (30–50%), which is one of the principal reasons for their low rate of microbial degradation, which is projected to take more than 50 years for complete degraded in the natural environment, and hundreds of years if discarded into the oceans, due to their lower temperature and oxygen availability. The enzymatic degradation occurs in two stages: adsorption of enzymes on the polymer surface, followed by hydro-peroxidation/hydrolysis of the bonds. The sources of plastic-degrading enzymes can be found in microorganisms from various environments as well as digestive intestine of some invertebrates. Microbial and enzymatic degradation of waste petro-plastics is a promising strategy for depolymerization of waste petro-plastics into polymer monomers for recycling, or to covert waste plastics into higher value bioproducts, such as biodegradable polymers via mineralization. The objective of this review is to outline the advances made in the microbial degradation of synthetic plastics and, overview the enzymes involved in biodegradation.

## Introduction

Petroleum-derived (petro-)plastics have many desirable characteristics. They are lightweight and have very stable chemical and physical properties, which makes them highly durable. Production methods are well established and very high capacity, resulting in very low cost. Consequently, they have become ubiquitous in the global economy (Barnes et al., 2009; Andrady, 2011; Hidalgo-Ruz et al., 2012). However, accumulation of petro-plastics wastes in the environment is now a major global problem because these materials are recalcitrant to natural biodegradation processes. As a result of their massive accumulation in municipal waste systems, micro- and nano-sized plastic particles are now ubiquitous in both terrestrial and aquatic ecosystems (Álvarez-Hernández et al., 2019; Bergmann et al., 2019). About 335 million tons of plastics were manufactured worldwide in 2016 (Plastics Europe, 2017). Synthetic plastics such as polyethylene terephthalate (PET), polyethylene (PE), polyurethane (PUR), polystyrene (PS), polypropylene (PP), and polyvinyl chloride (PVC) have been extensively utilized in a wide-range of industrial and domestic applications (Shah et al., 2008).

Environmental pollution by plastic waste was first reported in the 1970s (Carpenter and Smith, 1972). The growing amount of plastic waste has become a global concern. Despite increasing efforts to reduce the plastic waste by disposing off through segregated collection and recycling, a sizeable amount of plastic solid waste is still landfilled. From whole plastic production by 2017 (8,300 million tons), after recycling, incineration (energy recovery) of wastes and calculating in-use plastics in domestic; around 60% have been left in the environment including 95% in landfills and 5% in the oceans and other terrestrial areas (Ragaert et al., 2017). Plastic debris in the environment is degraded in nature by photo-, bio-, and thermo-oxidative depolymerization as well as friction (Barnes et al., 2009; Browne et al., 2011). Although biodegradation of these plastics is feasible in the natural environment, it can take long periods of time: from 50 to more than 100 years (Table 1).

**TABLE 1:** Selected properties of major synthetic thermoplastic polymers (Ojeda, 2013).

Polymer	Density (g/L)	Crystallinity (%)	Life span (years)
PET	1.35	0–50	450
LDPE	0.91–0.93	50	10–600
HDPE	0.94–0.97	70	>600
PS	1.03–1.09	0	50–80
PP	0.90–0.91	50	10–600
PVC	1.35–1.45	0	50–150

*PET, Polyethylene terephthalate; LDPE, Low density polyethylene; HDPE, High density polyethylene; PS, Polystyrene; PP, Polypropylene; PVC, Polyvinyl chloride.*

**FIGURE 1:**
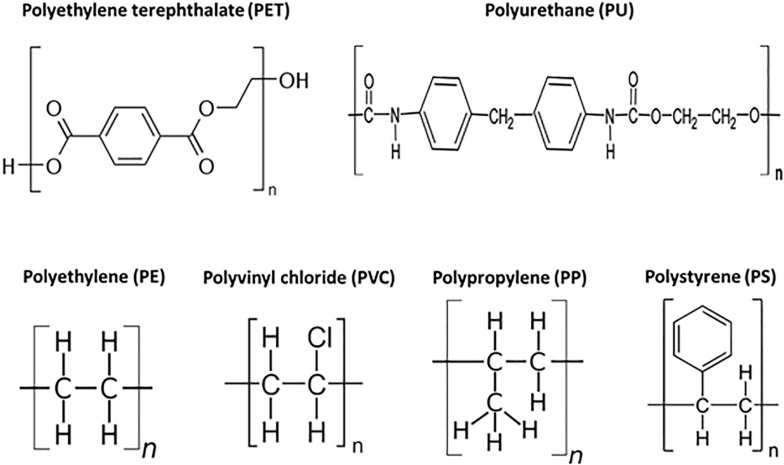
Structures of major commercial synthetic polymers.

Based on the degradation pathways, these synthetic plastics have been divided into two groups, plastics with a carbon-carbon backbone and plastics with heteroatoms in the main chain. PE, PS, PP, and PVC plastics have a backbone which is only built of carbon atoms (Figure 1). PET and PU have heteroatoms in the main chain (Figure 1). Several biodegradable aliphatic polyesters such as polyhydroxyalkanoate (PHA) and polylactic acid (PLA), were produced and may be used as alternatives to some petro-plastics (Tokiwa et al., 2009; Bhatia et al., 2019a, b). Polyhydroxyalkanoates (PHAs) have emerged as a sustainable choice due to their putative high biodegradability in different environments, biocompatibility, chemical diversity, their manufacture from renewable carbon resources, and release of non-polluting and non-toxic products after degradation (Bhatia et al., 2019a, b). PHAs are synthesized and accumulated by many prokaryotic microorganisms as storage compounds for carbon and energy when a major non-carboneous nutrient (e.g., nitrogen or phosphorus) is limiting. The accumulation of these polymers facilitates enhanced survival under environmental stress conditions. However, the most commonly used plastics are still synthetic polymers derived from petrochemical hydrocarbons (Geyer et al., 2017).

**FIGURE 2:**
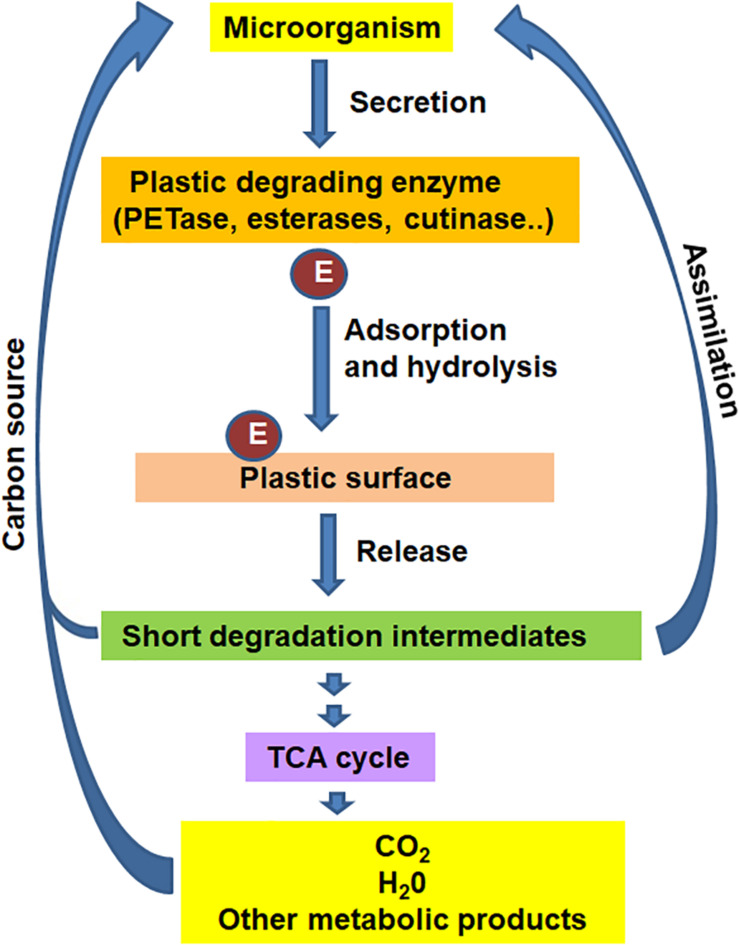
The general mechanism for biological degradation of plastics under aerobic conditions.

Degradation of plastics by microbial and/or enzymatic means (Figure 2) is a promising strategy to depolymerize waste petro-plastics into monomers for recycling, or mineralize them into carbon dioxide, water, and new biomass, with concomitant production of higher-value bioproducts (Grima et al., 2000; Montazer et al., 2019, 2020a). Biodegradation of plastics involves excretion of extracellular enzymes by the microorganism, attachment of enzyme to the surface of plastic, hydrolysis to short polymer intermediates, which are ultimately assimilated by microbial cells as carbon source to release CO_2_. Despite the fact that these plastics represent non-natural chemicals, several microorganisms capable of metabolizing these polymers have been identified in recent years. Over 90 microorganisms, including bacteria and fungi, have been known to degrade petroleum-based plastics (Jumaah, 2017) mostly *in vitro* condition.

**FIGURE 3:**
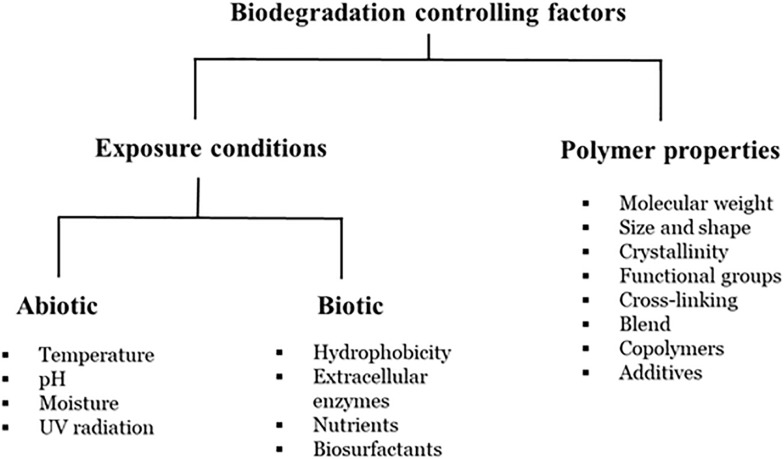
Factors affecting polymer biodegradation (adapted from Kijchavengkul and Auras, 2008).

Biodegradation is a complex process which is dependent on several factors, such as availability of a substrate, surface characteristics, morphology, molecular weight of the polymers (Figure 3) and therefore, an exact definition of biodegradation is lacking (Albertsson et al., 1987; Ammala et al., 2011; Harrison et al., 2018). Further, biodegradation has been measured by a wide-range of variables, including substrate weight loss, changes in the mechanical properties and/or the chemical structure of the polymer and the percentage of carbon dioxide emission. Early microbial biodegradation experiments attempted to demonstrate that microbial activity could result in changes in the physical characteristics of plastics, such as tensile strength, crystallinity, and water uptake (Pirt, 1980; Albertsson and Karlsson, 1990). The identification and genetic engineering of these plastic-degrading microorganisms and/or enzymes will provide an opportunity to improve plastic recycling and thereby reduce environmental plastic pollution by means of assimilation of plastic waste into carbon source or degradation of plastics waste into valuable alkane products via microbial biotechnology. The biodegradation mechanisms of petro-plastics are likely related to the types of bonds in the polymeric chains (since the active sites of related enzymes are individual for any specific bond). Thus, mechanisms of petro-plastic degradation can be classified into three groups: (i) Polymers with carbon back-bones; (ii) Polymers with ester-bond back-bones and side-chains; and (iii) Polymers with hetero/carbamate(urethane) bonds.

The present review outlines the recent advances made in the microbial degradation of synthetic plastics like polyethylene PE, polystyrene, polypropylene, polyvinyl chloride, PET, and PU and, overview the enzymes involved in biodegradation. Therefore, the study contributes to the existing knowledge in the field of microbial and/or enzymatic degradation of the synthetic plastics. Polymer degradation by microbial and enzymatic means is a promising strategy to convert plastic waste into carbon dioxide, polymer monomers, and possibly value-added compounds.

## Petro-Plastics With Carbon Back-Bones

Polyethylene, polypropylene, polystyrene, and polyvinyl chloride have a backbone which is solely made of carbon atoms. These polymers constitute the main packaging materials (Plastics Europe, 2017). UV-radiation and oxygen are the major initiation factors responsible for degradation of polymers with a carbon-carbon backbone, leading to fragmentation or chain scission. Photo-initiated oxidative degradation of PE, PP, and PS leads to reduced molecular weight and formation of carboxylic end groups, and UV-light initiates dechlorination in PVC polymers. The resulting smaller polymer fragments are susceptible to biodegradation and, abiotic degradation precedes biodegradation. Plastics Additives (1998) demonstrated that only ∼0.1% per year of the carbon that makes up the polymer is converted into CO_2_ by biodegradation under optimum laboratory conditions.

### Polyethylene

#### PE Degrading Microorganisms

PE is composed of a linear chain of carbons held together by hydrogen bonds (Figure 1). PE usually has a semi-crystalline structure and is extremely resistant to biodegradation. Based on different manufacturing processes and subsequently different arrangements of the linear chains, PE polymers can have different densities and 3-dimensional and physical structures, low molecular weight polyethylene (LMWPE), linear low-density polyethylene (LLDPE), low-density polyethylene (LDPE), and high-density polyethylene (HDPE).

Biodegradation of the PE plastic wastes by microbial isolates has been a major topic of research (Nowak et al., 2011; Kyaw et al., 2012; Gajendiran et al., 2016; Sen and Raut, 2016). Microorganisms capable of hydrolyzing PE have been isolated from soil, sea water, compost and activated sludge (Montazer et al., 2020a). Bacteria in the gut of the greater wax worm, *Galleria melonella* have been found capable of hydrolyzing polyethylene (PE) (Yang et al., 2014; Bombelli et al., 2017; Cassone et al., 2020). Besides PET, biotechnological conversion of PE through pyrolysis and transformation or direct transformation of PE to polyhydroxyalkanoate in cells, has been reported in different Pseudomonads (Ward et al., 2006; Kenny et al., 2008; Guzik et al., 2014; Montazer et al., 2019).

Bacterial species, such as *Bacillus* spp. (Sudhakar et al., 2008; Abrusci et al., 2013), *Rhodococcus* spp. (Bonhomme et al., 2003; Gilan et al., 2004; Fontanella et al., 2010), and *Pseudomonas* spp. (Rajandas et al., 2012), and fungi, such as *Aspergillus* and *Fusarium* (Hasan et al., 2007; Sahebnazar et al., 2010), were shown to depolymerize PE after some forms of pretreatment such as Ultraviolet (UV) and/or thermal treatments, which render the carbon chains of polymer sensitive to biodegradation (Ammala et al., 2011). Microbial degradation of untreated PE has been reported in few others such as *Pseudomonas putida* IRN22, *Acinetobacter pittii* IRN19, *Micrococcus luteus* IRN20 (Montazer et al., 2019) and different species of *Pseudomonas*, *Pseudomonas aeruginosa* PAO1, *P. aeruginosa* ATCC, *P. putida*, and *Pseudomonas syringae* (Kyaw et al., 2012), *Pseudomonas* sp. E4 (Yoon et al., 2012), and bacterial strains from the genera *Comamonas*, *Delftia*, and *Stenotrophomonas* (Peixoto et al., 2017).

#### Mechanism of PE Degradation

Aerobic biodegradation of PE by bacteria occurs in four stages: (1) Biodeterioration, which is defined as formation of carbonyl-groups by the action of oxidative enzymes released by microorganisms or induced by exterior agents, like sunlight (ultra-violet) exposure. Subsequent oxidation reduces the number of carbonyl-groups and generates carboxylic acids; (2) Biofragmentation, which involves hydrolysis and/or fragmentation of the polymer carbon chains and the release of intermediate products, mediated by enzymes secreted by microorganisms; (3) Bioassimilation, whereby small hydrocarbon fragments released by biofragmentation are taken-up and metabolized by bacteria or fungi. and (4) Mineralization, transfer the hydrolysis products within the cell wall, intracellular conversion of hydrolysis products to microbial biomass with the associated release of carbon dioxide and water excreted out the cell. Though several groups have reported on biodeterioration and biofragmentation (Albertsson and Karlsson, 1990; Ammala et al., 2011), studies on bioassimilation and complete mineralization of PE are very limited (Yang et al., 2014; Sen and Raut, 2016; Montazer et al., 2019).

Montazer et al. (2019) reported the biofragmentation and bioassimilation of LDPE into biomass by the bacterial species, *Pseudomonas putida* IRN22, *P. putida* LS46, *Acinetobacter pittii* IRN19, and *Micrococcus luteus* IRN20. These bacterial species were able to utilize the untreated petroleum-derived LDPE as a sole source of carbon and energy for growth and generate alkane hydrolysis products as well as accumulate biodegradable polymers in the form of short chain length (scl-) and medium chain length (mcl-) polyhydroxyalkanoates (Montazer et al., 2019). While, *A. calcoaceticus* and *P. aeruginosa* were found to metabolize only alkane hydrocarbons and not LPDE (Guzik et al., 2014).

Low-density polyethylene is the most abundant plastic waste discarded in landfills in the form of plastic bags (69.13%). LDPE is mostly amorphous, with short branches (10–30 CH_3_ per 1,000 carbon atoms) and composed of one or more comonomers, such as 1-butene, 1-hexene, and 1-octene. This branching system makes LDPE chains more accessible and the tertiary carbon atoms at the branch sites more susceptible to attack. The physical arrangement of the polymer chains in LDPE and a lower content of vinylidene defects, which have been shown to be directly correlated with oxidization of the polymer makes it more biodegradable than High-density polyethylene (HDPE). Further, the molar mass of HPDE is much higher, possibly making it more difficult for microorganisms and their oxidizing enzymes to access the polymer chains (Sudhakar et al., 2008; Fontanella et al., 2010). Few comparison studies have been made on the biodegradability of various pre-treated polyethylene materials, including, LDPE, HDPE, and linear low-density polyethylene (LLDPE) films of different thickness by *Rhodococcus rhodochrous*, which is one of the most efficient bacteria for PE biodegradation (Fontanella et al., 2010). Structural variations in PE polymers formed during polymerization and subsequent processing, such as unsaturated carbon–carbon double bonds, carbonyl groups, and hydroperoxide groups (Ojeda et al., 2011) have been shown to be consumed first by the bacteria resulting in rapid growth.

Many of the species known to degrade PE are capable of hydrolyzing and metabolize linear *n*-alkanes, like paraffin molecules (e.g., C_44_H_90_, Mw 618) (Haines and Alexander, 1974). Alkane hydroxylases (AHs) are the key enzymes involved in aerobic degradation of alkanes by bacteria. The first step involves hydroxylation of C-C bonds to release primary or secondary alcohols, which are oxidized to ketones or aldehydes, and subsequently to hydrophilic carboxylic acids (Alvarez, 2003; Watanabe et al., 2003). Microbial oxidation reduces the number of carbonyl-groups due to the formation of carboxylic acids. Carboxylated *n*-alkanes are analogous to fatty acids, which are catabolized by bacteria via the β-oxidation system pathway (Restrepo-Flórez et al., 2014). Usha et al. (2011) and Yoon et al. (2012) have demonstrated the microbial oxidation of *n*-alkanes by bacteria via the β-oxidation pathway and subsequently to the tricarboxylic cycle. The oxidation products released by the action of enzymes in the process may be absorbed by microbial cells where they are catabolized.

Eyheraguibel et al. (2017) revealed that extracellular mechanisms leading to enzymatic oxidation and hydrolysis of chains of PE polymers are also significant They documented oligomers production with maximum 55 carbons (molar mass of 105–850 g/mol) from PE films that adsorbed by bacteria after 240 days of incubation. AHs forms the key enzyme in the alkane hydroxylase system pathway, which are involved in PE degradation in β-oxidation pathway and known to degrade linear alkanes (Jeon and Kim, 2015). The most important enzymes of interest in the alkane hydroxylase system are the monoxygenases. The number and types of AHs vary greatly in different bacteria, which itself differs in the amount of carbon in the alkane chains (Jeon and Kim, 2016). The *Rhodococcus* sp. TMP2 genome encodes 5 AHs (*alk*B1, *alk*B2, *alk*B3, *alk*B4, and *alk*B5) while the *P. aeruginosa* genome encodes two AHs: *alk*B1 and *alk*B2 (Takei et al., 2008). The Alkane hydroxylase system has also been well studied in *P. putida* GPo1, where the enzyme is involved in the hydroxylation of the terminal carbon-first step of the n-alkane oxidation pathway (Rojo, 2009). Yoon et al. (2012) have shown that AlkB enzyme of *P. aeruginosa* strain E7 played a central role in the mineralization and thus, biodegradation of LMWPE into CO_2_ (Yoon et al., 2012). The *alkB* gene was cloned in *Pseudomonas* sp. E4, and the AlkB enzyme expressed from the recombinant strain participated in the early stage of LMWPE biodegradation, in the absence of the other specific enzymes like rubredoxin and rubredoxin reductase. A class of multi-copper enzymes, known as Laccase enzymes (phenol oxidases), expressed by *Rodococcus rubber*, have also been shown to play an important role in PE biodegradation (Santo et al., 2013).

#### New Approaches in PE Biodegradation by Invertebrates

Recent studies have shown that the digestive tracts (gut) of some insects function as bioreactors, with digestive enzymes and gut microbiomes that appear to contribute to and accelerate the biodegradation rate of some recalcitrant plastics. Riudavets et al. (2007) first observed that some insects are able to chew and penetrate PE packaging. Yang et al. (2014) isolated LDPE-degrading bacterial strains, *Enterobacter asburiae* YT1 and *Bacillus* sp. YP1 from the gut of *Plodia interpunctella* (Indianmeal moth). Bacterial isolates identified from the gut of *P. interpunctella* were able to degrade approximately 6–10% of PE films and released 12 water-soluble products. Yang et al. (2015) reported the complete genome sequence of *Bacillus* sp. strain YP1, one of isolates from the gut of *P. interpunctella* larvae that was implicated in polyethylene depolymerization and biodegradation. Bombelli et al. (2017) realized the extraordinary ability of larvae of the greater wax moths, *Galleria mellonella*, to rapidly degrade polyethylene, while producing ethylene glycol (EG). In 2019, *Enterobacter* sp. D1 was isolated from the gut of *G. mellonella* by Ren et al. (2019). They confirmed physical changes in the PE-film after treatment, and suggested these physical changes were the result of oxidation reactions caused by the bacteria.

Cassone et al. (2020) assessed the biodegradation of PE by *G. mellonella* larvae and observed similar results. A diet of LDPE alone enabled subsistence of *G. mellonella* larvae, but was insufficient for growth. These results suppose real biodegradation was not occurred during passing the PE through the gut’s larva, but the first biodeterioration or minor oxidation may be taken place resulting in changing in physical properties rather than chemical ones. Billen et al. (2020) showed chewing and ingesting of polyethylene by *T. molitor* larvae created holes and reduced the size of polyethylene films, but digestion was not explicitly confirmed. They suggested that the intestinal microbiomes of these insect larvae play key role in the initial short-term biodegradation process, which occurs rapidly within the larval insect gut. However, the insect gut microbiome alone is not sufficient for this initial rapid biodegradation of PE. Rather, both the insect digestive system and the larval gut microbiome are required to achieve accelerated biodegradation of the PE polymers.

In recent years, biodegradation of other petro-plastics, like PE and PS, insect larvae such as Yellow Mealworms (*Tenebrio molitor*) (Billen et al., 2020), Dark Mealworms (*Tenebrio obscurus*) (Brandon et al., 2018; Peng et al., 2019), Superworms (*Zophobas atratus*) (Peng et al., 2020b), Lesser Waxworms (*Achroia Grisella*) (Kundungal et al., 2019), and by snails (*Achatina fulita*) (Song et al., 2020), have been reported. Yin et al. (2020) isolated two LDPE-degrading strains from the gut of *T. molitor* larvae, *Acinetobacter* sp. strain NyZ450 and *Bacillus* sp. strain NyZ451. The cells of both strains can depolymerize LDPE but did not grow on it. Their co-culture grew on LDPE and removed LDPE mulching films by 18% over 30 days. This suggests that biodegradation of LDPE requires multiple microbes. Yang et al. (2021) reported that antibiotic suppression did not stop LDPE depolymerization in the gut of *Tenebrio molitor* larvae indicating that the digestive enzymes of the larvae are capable of breaking down LDPE.

Despite high rates of biodegradation of PE by live macro-organisms, there are a number of drawbacks that may limit the use of insect larvae as a waste management strategy for petro-plastics like polyethylene. These limitations include: (i) the need to sustain insect cultures to produce the larvae that feed on PE; (ii) the potentially high cost of maintaining these cultures; and (iii) generation of microplastics that may contribute to environmental problems, due to incomplete degradation and lack of mineralization Instead, finding new isolates of bacteria and/or fungi with the ability to degrade PE, and understanding the exact mechanisms of biodegradation pathways, may be more efficient in developing of new methods of PE waste management (Billen et al., 2020; Montazer et al., 2020b).

### Polystyrene

Polystyrene (PS) has been the most abundant plastics produced worldwide and largely manufactured into packaging materials for food and disposable dishware (Plastics Europe, 2017). Like other synthetic plastics, PS is extensively used because of its good mechanical properties and relatively low cost. PS is commonly used in packaging foam, food containers, construction materials (insulation), cassette boxes, compact disks, disposable cups, plates, and cutleries. There are approximately 21 million tons of PS produced worldwide in 2013 (Yang et al., 2015a, b). PS has been grouped into four types of product based on its different applications, General purpose polystyrene (GPPS)/oriented polystyrene (OPS), high impact polystyrene (HIPS), PS foam, and expanded polystyrene (EPS) foam (Ho et al., 2018). PS are extremely stable polymers with high molecular weight and strong hydrophobic character, which makes these polymers highly resistant to biodegradation (Albertsson and Karlsson, 1993; Ho et al., 2018). The carbon–carbon backbone in PS is highly resistant to enzymatic cleavage by oxidation–reduction process (Goldman, 2010).

The biodegradation of PS by several microbes and/or microbial enzymes has been demonstrated (Table 2). PS materials used in these studies range from pure PS or modified PS to PS blended with other polymers. A pure strain of *P. aeruginosa* degraded the modified PS (Shimpi et al., 2012), and *Curvularia* species was investigated for degradation of atactic PS, without any pretreatment (Motta et al., 2009). The modified PSs used have been PS/PLA composites, and PS/PLA/organically modified montmorillonite (OMMT) blends (soft phyllosilicate minerals that usually form in microscopic crystals, forming clays) (Shimpi et al., 2012). Pure strains of the actinomycete, *Rhodococcus ruber* have been shown to degrade three forms of PS, pure standard PS flakes, PS powder, and ELISA 96-well microtiter plates produced from pure PS (Mor and Sivan, 2008; Santo et al., 2013). Ward et al. (2006) reported that the *P. putida* CA-3 (NCIMB 41162) uses styrene oil after pyrolysis as the sole source of carbon and energy to form PHAs. Several investigators have shown that the rate of biodegradation of PS foams and films can be improved by using the polymer-starch blends, which accelerates the structural molecular changes (Jasso et al., 1998; Schlemmer et al., 2009; Pushpadass et al., 2010; Nikolic et al., 2013).

**TABLE 2:** Microorganisms capable of recalcitrant petro-plastics degradation.

Examined polymer (polymer under examination)	Species	Source	Cultivation conditions	Polymer degradation		References
	
				*In vitro* conditions	Degradation efficiency	
*Polystyrene*	*Pseudomonas* sp.	Soil samples from plastic dump yard	Mineral medium with 0.85% NaCl and HIPS film at 30°C, 150 rpm	30 days incubation at 30°C	>10% weight loss	Mohan et al., 2016
	*Bacillus* sp.				23.7% weight loss	
	*Pseudomonas aeruginosa*	Degraded polymer nanocomp-osite	NB medium at 30°C for 24 h	28 days incubation at 30°C in MSM	9.9% degradation at 10 and 25% PS: PLA composites	Shimpi et al., 2012
	*Pseudomonas putida* CA-3	Industrial bioreactor isolate	E2 mineral medium with 67 mg nitrogen/l and 9.5 mg/l styrene oil at 30°C, 200 rpm for 24 h	48 h of fermentation at 30°C, 500 rpm	A single pyrolysis run and four fermentation runs resulted in the conversion of 64 g of polystyrene to 6.4 g of PHA	Ward et al., 2006
	*Curvularia* sp.	Soil samples	Sabouraud’s broth at 25°C for 13 days	9 weeks incubation at 25°C in Sabouraud’s agar embedded with Ecoflex	Microscopic examination showed adherence and penetrance to the polymer	Motta et al., 2009
	*Rhodococcus ruber* *Enterobacter* sp. *Citrobacter sedlakii* *Alcaligenes* sp. *Brevundimonas diminuta*	Soil samples	NB medium at 35°C, 120 rpm for 10–14 days	8 weeks incubation at 35°C in synthetic medium	0.8% weight loss	Mor and Sivan, 2008
	*Exiguobacterium* sp. strain YT2	Degraded plastic waste	MSM with e-plastic film at 30°C, 150 rpm for 2 weeks	30 days incubation at 30°C, 150 rpm in mineral medium	12.4% weight loss	Sekhar et al., 2016
*Polypropylene*	*Pseudomonas* *Vibrio* *Aspergillus niger*	Plastic dumping site	Mineral medium (B7) with 0.05% glucose and 0.05% sodium lactate at 30°C	175 days incubation at neutral pH and 30°C	60% weight loss	Cacciari et al., 1993
	*Bacillus flexus*	Plastic dumping site	Minimal media with 0.25% glucose at 37°C	365 days incubation at neutral pH and at 35–37°C, 180 rpm	2.5% weight loss	Arkatkar et al., 2010
	*Bacillus cereus*	Mangrove sediments	Mineral salt medium at 29°C	40 days incubation at 33°C, 150 rpm	12% weight loss	Auta et al., 2017
	*Sporosarcina globispora*				11% weight loss	
	*Bacillus* sp.	Municipal compost waste	Minimal media at 37°C	15 days incubation at 37°C, 120 rpm	10–12% weight loss	Jain et al. (2018)
*Polyvinyl chloride*	White rot fungi	Department of Agriculture Forest Products Lab. Madison, Wisconsin, United States	Liquid medium at 30°C, pH 4.5 and 150 rpm for 7 days	30 days incubation at 30°C	Not specified (intrinsic viscosity of the degraded polymer films decreased)	Klrbas et al., 1999
	*Trichocladium* sp. *Chaetomium* sp.	Soil samples	ISP2 (Difco) medium at 25°C for 7 days	90 and 180 days incubation at 30°C	1 or 0.4% after 3 or 6 months, respectively	Kaczmarek and Bajer, 2007
	*Pseudomonas citronellolis* *Bacillus flexus*	Leibniz Institute DSMZ-German Collection of Microorganisms and Cell Cultures (Germany)	MSM with 20 g/l glucose at 30°C, 150 rpm	90 days incubation at 30 and 37°C, 150 rpm	19% after 30 days incubation	Giacomucci et al., 2019
	*Pseudomonas aeruginosa*	Activated sludge (waste water treatment plant)	MSM with (NH_4_)_2_SO_4_ 0.67 g/liter at 23°C, 150 rpm	24 days incubation 23°C	VC concentrations as high as 7.3 mM were biodegraded	Verce et al., 2000
	*Pseudomonas otitidis* *Bacillus cereus* *Acanthopleuro-bacter pedis* *Aspergillus fumigatus* *Aspergillus niger* *Aspergillus sydowii* *Phanerochaete chrysosporium* *Lentinus tigrinus*	Soil samples	MSM at 30°C, 150 rpm	90 days incubation at 30°C	Clear surface aberrations and disintegration in PVC films. Significant decrease in the molecular weight of film from 80,275 to 78,866 Da	
*Polyethylene terephthalate*	*Ideonella sakaiensis*	PET recycling factory	NB medium with PET at 30°C	42 days incubation at 30°C	Almost complete degradation achieved	Yoshida et al., 2016
	*Bacillus subtilis*	TU-Graz culture collection	NB medium at 30° C, 125 rpm	24 h incubation at 30°C and pH 7.0	Not specified (degradation products determined by LC-MS/MS analysis)	Ribitsch et al., 2011
	*Pseudomonas putida* GO16	National Collection of Industrial, Food and Marine Bacteria (NCIMB)	MSM medium with sodium terephthalate produced from a PET pyrolysis product and waste glycerol from biodiesel at 30°C, 200 rpm for 24 h	48 h incubation at 30°C	PHA productivity (g/l/h) 1.8- to 2.2-fold	Kenny et al., 2012
	*Thermomonospora fusca*	6-month-old mature compost from green waste (compost plant)	MSM medium at 55°C for 14 days	14 days incubation at 55°C on MSV agar	Degradation rates of 20 mg/week cm^–2^	Kleeberg et al., 1998
	*Thermobifida alba* AHK119	Composted polyester films. IPOD of AIST, Tsukuba, Japan)	LB medium containing the hydrolyzed polymer suspension at 50°C for 3 days	14 days incubation at 50°C	Zone of clearance was observed	Hu et al., 2010
	*Thermomonospora curvata*	Composts containing plant materials	MSM medium at 50°C at a wide range of pH from 7.5 to 11 for 3 days	7 days incubation at pH 8.5 and 50°C, 55 and 60°C	At 50°C, hydrolysis rate 3.3 × 10^–3^ min^–1^	Wei et al., 2014b
	*Thermobifida halotolerans*	German Resource Centre for Biological Material (DSMZ, Germany)	LB medium at 37°C, 160 rpm for 24 h	2 h incubation at 50°C, 350 rpm and pH 7.0	The amount of MHET and TA released were around 19.8 and 21.5 mmol/mol of enzyme, respectively	Ribitsch et al., 2012a
	*Saccharomonospora viridis*	Compost (Okayama, Japan) IPOD of the NITE	LB medium at 50°C under shaking for 12–16 h	3 days incubation at 63°C under shaking	13.5% weight loss for PET-GF and 27.0% for PET-S	Kawai et al., 2014
*Polyurethane*	*Pseudomonas fluorescens*	National Research Laboratory, Washington D.C.	YES medium containing Impranil at 30°C for 5 days	24 h incubation at 30°C using polyurethane plates	Zone of clearance was observed	Howard and Blake, 1998
	*Pseudomonas chlororaphis*	Microbial consortium from the Naval Research Laboratory, Washington, DC, United States	LB medium at 30°C, 180 rpm	6 h incubation at 23°C on plates with Impranil	Zone of clearance was observed	Stern and Howard, 2000
	*Exophiala jeanselmei*	Soil samples from a polyurethane factory in Japan	Difco Sabouraud liquid medium at 25°C for 6–8 days	7 days incubation at 25°C with tolylcarbamate compounds that resemble the urethane segments in TDI-based polyurethanes	Able to hydrolyze the urethane in tolylcarbamate compounds	Owen et al., 1996
	*Aureobasidium pullulans* *Cladosporium* sp. *Curvularia senegalensis* *Fusarium solani*	Garden soil near Washington, D.C.	YES medium containing Impranil at 25°C for 6 days	Several days to weeks incubation at 25°C in YES-PG agar plates	Zone of clearance was observed	Crabbe et al., 1994
	*Pseudomonas aeruginosa* *Corynebacterium* sp.	River mud enriched with various platicisers	MSM with 1% yeast extract	12 weeks incubation at 25°C in MSM medium with yeast extract	9.30 + 1.32 %weight loss 15.77 ± 3.5 %weight loss	Kay et al., 1991
	*Comamonas acidovorans*	Soil samples of Tsukuba City in Japan	Basal mineral medium with polyurethane at 30°C, 120 rpm for 14 days	7 days incubation with PUR films at 30°C	About 48% of the added PUR was degraded	Nakajima-Kambe et al., 1995
	*Acinetobacter calcoaceticus* *Arthrobacter globiformis*	Oil-contaminated Connecticut soil	Tripticase soy broth at 30°C, 150 rpm for 24 h	10 days incubation at 30°C in MSM with polyurethane painted aluminum coupons	Release of pigment from the polyurethane coatings into the broth was observed in 192 h	El-Sayed et al., 1996
	*Bacillus subtilis*	Soil sample from a mecocosm study	YES medium at at 30°C, 150 rpm for 12 h	24 h incubation at 30°C on a LB agar plate with Impranil	Zone of clearance was observed	Rowe and Howard, 2002

*HIPS, High impact polystyrene; PHA, Polyhydroxyalkanoate; NB, Nutrient broth; MSM, Mineral salt medium; VC, Vinyl chloride; Leibniz Institute DSMZ, Leibniz-Instut Deutsche Sammlung von Mikroorganismen und Zellkulturen GmbH; MSM Mineral Salt Medium; NB, Nutrient broth; LB, Luria-Bertani (LB); MSM, Mineral Salt Medium; TU-Graz, Technische Universität Graz; IPOD, International Patent Organism Depositary; NITE, National Institute of Technology and Evaluation; PET-GF, amorphous polyethylene terephthalate (0.25-mm-thick film); PET-S, polyethylene terephthalate package (approximately 0.6-mm-thick); Leibniz Institute DSMZ, Leibniz-Instut Deutsche Sammlung von Mikroorganismen und Zellkulturen GmbH; MHET, mono-2-hydroxyethyl terephthalate; TA, Terephthalic acid; AIST, National Institute of Advanced Industrial Science and Technology; YES, Yeast-extract salts medium; YES-PG, Yeast-extract salts medium with 3 mg/ml of Impranil and 4 mg/ml of gelatin.*

Besides using starch as copolymer, the addition of prooxidants such as metal salt (iron, cobalt, and manganese) to increase biodegradation have also been investigated. It has been found that trace amounts of metals such as Co, Mn, Fe, Cu, and Ni, significantly increase the rate of oxidative degradation (Gorghiu et al., 2004). Prooxidants facilitates cleavage of molecules into smaller fragments containing hydrophilic oxygenated groups that can be easily biodegraded by microorganism in the environment (Shang et al., 2003). Ojeda et al. (2009) reported improved biodegradability of foamed PS when Co and Mn based prooxidant additives were used.

Styrene itself can be used as a carbon source for growth by some microorganisms (Sielicki et al., 1978; Kaplan et al., 1979; Mor and Sivan, 2008; Motta et al., 2009). *R. ruber* has been reported to form biofilms on PS and partially degrade it (Mor and Sivan, 2008). Styrene catabolism involves the oxidation of styrene to form phenylacetate, which is then converted via the TCA cycle (Di Gennaro et al., 1999). Similar to PE, both laccase and oxidoreductases have been shown to be involved in the biodegradation of PS, such as the AlkB family hydroxylases and hydroquinone peroxidase (Nakamiya et al., 1997; Jeon and Kim, 2015). Xu et al. (2019) has demonstrated the bio-catalytic mechanism of PE and PS degradation by oxidoreductase using quantum mechanism calculations, with the P450 monooxygenase involved in a typical saturated carbon-carbon bone cleavage reaction (Matthews et al., 2017).

Biodegradation of polystyrene foam (or Styrofoam) has been reported intensively during last 5 years. Yang et al. (2015a, b) found that the larvae of *Tenebrio molitor* are capable of depolymerizing and mineralizing PS to H_2_O and CO_2_ within 24 h, and the degradation is gut microbe dependent based on antibiotic suppression test. The ubiquity of gut-microbe dependent depolymerization and biodegradation of PS in *T. molitor* has been confirmed by multiple researchers (Yang et al., 2018a, b, 2021). The same PS biodegradation pattern has also been observed in the members of darkling beetles including *Tenebrio obscurus* (Peng et al., 2019) and *Zophobas atratus* (Peng et al., 2020b). PS-degrading bacterial cultures of *Exiguobacterium* sp. strain TY2 was isolated from the gut of *T. molitor* larvae (Yang et al., 2015b) while *Pseudomonas aeruginosa* strain DSM 50071 (Kim et al., 2020) was isolated from *Z. atratus*. The mass reduction rates by the bacterial cultures were much slower than that occurred in the gut of the larvae, suggesting that the synergetic biodegradation occurs in the digestive systems. In addition, PS depolymerization with limited extent pattern was also observed in the digestive intestines of land snails, *Achatina fulica* (Song et al., 2020) and larvae of *Galleria mellonella* (Lou et al., 2020). Antibiotic suppression with oxytetracycline did not stop PS depolymerization in the snails, suggesting the presence of PS-degrading enzyme(s).

### Polypropylene

Polypropylene (PP), expressed as C_n_H_2n_, is also the most widely used linear hydrocarbon polymers among the synthetic polymers. PP has a methyl-group in place of one of the hydrogens present in PE on every other carbon, which gives rise to the presence of three stereoisomeric forms namely, atactic, isotactic, and syndiotactic (Baker and Mead, 2002). This polymer was first synthesized by J. Paul Hogan and Robert L. Banks in 1951 with propylene as the monomer (Stinson, 1987). Metallocene catalysts have also been used for its synthesis. Its properties are similar to polyethylene, but it is slightly harder and more heat resistant and has a high chemical resistance Polypropylene is the second-most widely produced commodity plastics (after polyethylene) and it is often used in packaging and labeling. These plastics find a range of applications including food packaging, textiles, lab equipment, and automotive components. PP are grouped as polyolefins together with PE, defined as inert materials not prone to microbial attack because of the hydrophobic backbones composed of long carbon chains, high molecular weight (from 10,000 to 40,000 g/mol) and the added antioxidants and stabilizers during their manufacture which prevents polyolefins from atmospheric oxidation (Zheng and Yanful, 2005). However, substitution of methyl in place of hydrogen in the β-position makes it more resistant to microbial attack (Zuchowska et al., 1998, 1999).

Very few studies of PP biodegradation have been reported (Table 2). Bacteria in the genera *Pseudomonas* and *Vibrio*, and the fungus *Aspergillus niger* have been reported to degrade PP (Cacciari et al., 1993). Most of the studies have been carried out using pretreated PP. The pretreatment techniques involved γ-irradiation (Iwamoto and Tokiwa, 1994), UV-irradiation (Huang et al., 2005; Kaczmarek et al., 2005; Sameh et al., 2006), or thermal treatment (Ramis et al., 2004) and have been shown to reduce the hydrophobicity of the polymer or introduces groups such as C=O or –OH, which are more susceptible to degradation. Formation of new groups (carbonyl and hydroxyl) and a decrease in viscosity have been observed during the degradation process (Iwamoto and Tokiwa, 1994; Sameh et al., 2006). *Bacillus flexus* has been shown to biodegrade UV-pretreated PP (Arkatkar et al., 2010). Biodegradation of polypropylene have been improved by using polymer blends with carbohydrates, starch or cellulose blends like that reported for polyethylene and polystyrene. The use of the blends facilitates adhesion of the microorganisms to the surface of the polymer and acts as a co-metabolite (Cacciari et al., 1993; Zuchowska et al., 1998; Ramis et al., 2004; Kaczmarek et al., 2005; Morancho et al., 2006). Biodegradation of Polycaprolactone (PCL) blended PP has also been demonstrated using lipase since lipase is well known to degrade the ester linkages of PCL (Weiland et al., 1995).

### Polyvyinylchloride

Poly(vinyl chloride) (PVC) is a synthetic polymer well known for many years and are widely used in its rigid or plasticized form (Braun, 2004). However, the pollution caused by waste PVC-based plastics is a serious problem. Many studies have reported on the thermal and photo degradation of PVC (Braun, 1975; Owen, 1976, 2012; Decker, 1984; Seppala et al., 1991). Torikai and Hasegawa (1999) reported accelerated photodegradation of PVC when exposed to short-wavelength radiation. However, biodegradation of PVC has been attempted in very few studies (Klrbas et al., 1999; Kaczmarek and Bajer, 2007; Ali et al., 2014; Giacomucci et al., 2019) and shown by very few microorganisms (Table 2). White rot fungi in the Basidiomycotina were reported to biodegrade low molecular weight PVC when subjected to nutrient (nitrogen, carbon or sulfur) limiting conditions (Klrbas et al., 1999).

The ability of the white rot fungi to degrade the organopollutants to carbon dioxide, has been shown to be dependent on a nonspecific, non-stereo-selective lignin degrading system. Lignin is a complex heteropolymer and is probably the most-difficult-to degrade naturally occurring organic compound. The lignin degrading system includes a group of peroxidases known as Ligninases, which catalyze the initial oxidative depolymerization of lignin polymers (Bumpus and Aust, 1987; Bumpus et al., 1988; Shah et al., 1992). The growth of *Trichocladium* sp. and *Chaetomium* sp. was accelerated in the presence of cellulose with PVC as a carbon source, suggesting co-meatbolims of PVC with the cellulose (Kaczmarek and Bajer, 2007). Also, additives introduced into plastics such as plasticizers, contribute to fungal nutrients for growth.

*Pseudomonas citronellolis* and *Bacillus flexus* have also been found to biodegrade PVC film, with high depolymerizing activity toward PVC additives relative to the PVC polymer chains (Giacomucci et al., 2019). These strains have been shown to form dense biofilm on the plastic film surface and cause a decrease in the mean molecular weight of the PVC film. A dense biofilm and a decrease in mean molecular weight (Mn) has been reported as proof of polymer chain biodegradation (Das et al., 2012; Wilkes and Aristilde, 2017; Syranidou et al., 2017; Ahmed et al., 2018). Decreases in molecular weight can be attributed to the action of exocellular enzymes released into the culture medium, which causes the hydrolysis of polymers at the ends of backbone chains as well as within the chains (Wilkes and Aristilde, 2017). *Pseudomonas putida* strain AJ was reported to use vinyl chloride monomers as carbon source for growth (Verce et al., 2000). The composting and biodegradation processes of PVC are still controversial because of the possible formation of degradation products containing chlorine, which are not neutral for the environment.

Peng et al. (2020a) found that *T. molito*r larvae are capable of depolymerizing and biodegrading PVC materials rapidly to organic chlorinated intermediates but mineralization extent was very limited with only 2.9% of PVC conversion to chloride. The depolymerization stopped when gut microbes were suppressed with antibiotic gentamicin, indicating gut-microbial dependence, similar to observations recorded for PS degradation by *T. molitor* larvae.

## Petro-Plastics With Ester-Bond Back-Bones and Side-Chains

Polyethylene terephthalate (PET) and polyurethane (PU) plastics have heteroatoms in the main chain. Plastics composed of polymers with carbon and hetero atoms in the main chain have improved thermal stability relative to polymers with only carbon backbone (Venkatachalam et al., 2012). These polymers are susceptible to hydrolytic attack of e.g., ester or amide bonds (Muller et al., 2001). Plastics with heteroatoms in the main chain can be degraded by photo-oxidation, hydrolysis and biodegradation (Muller et al., 2001). This results in the formation of smaller fragments and carboxylic end groups.

### Polyethylene Terephthalate

#### PET Degrading Microorganisms

Polyethylene terephthalate is one of the major synthetic petro-plastics (Figure 1) that is produced in very large amounts globally. Its worldwide production accounted to 56 million tons in 2013 (Neufeld et al., 2016). PET consists of aromatic polyesters with high glass transition temperatures (Tg) of approximately 75–80°C in air. However, Tg decreases to 60–65°C in aqueous solution (Kawai et al., 2014). At temperatures above the Tg, the amorphous regions of PET become flexible and more accessible to microbial degradation and/or enzymatic attack. With polymer degradation, a decrease in Tg was observed as a result of reduction in the average chain length, due to the higher motility of shorter chains (Odusanya et al., 2013).

PET is used in a wide-variety of applications, such as in manufacturing bottles, containers, textile fibers, and films. PET polymers differ in crystallinities based on its usage. While, most PET used for manufacturing textiles and bottles has high crystallinity (30–40%), PET used for packaging has less crystallinity (approximately 8%) (Kawai et al., 2014). Commercially available low-crystalline PET (PET-GF) has approximately 6–7% crystallinity (Ronqvist et al., 2009; Kawai et al., 2014).

Although most biodegradable plastics are polyesters [e.g., polyhydroxyalkanoate, PCL, polybutylene succinate, polybutylene succinate-co-adipate, and poly(butylene adipate-co-terephthalate) (PBAT)], PET, which is also a polyester, but is considered to be recalcitrant to biodegradation (Marques-Calvo et al., 2006). However, several microorganisms capable of metabolizing these polymers have been identified in recent years (Table 2). These include the bacterium *Ideonella sakaiensis* 201-F6, is able to depolymerize PET polymers and utilize the terephthalate subunits as a carbon and energy source for metabolism and growth (Yoshida et al., 2016). Biotechnological conversion of PET through pyrolysis and conversion to polyhydroxyalkanoate has been demonstrated using different Pseudomonads (Ward et al., 2006; Kenny et al., 2008; Guzik et al., 2014). Pyrolysis of PET resulted in terephthalate, which was used as feedstock for *P. putida* GO16 (Kenny et al., 2012). The degradation rate of PET films depends on the crystallinity, purity of films and orientation of the polymer chains. The degradation of the commercially available standard PET film (pure and amorphous PET) at 50°C was found significantly lower (approximately 5%), however, the degradation increased with high temperatures (55, 60, and 65°C) to more than 30% (Oda et al., 2018).

#### Mechanism of PET Biodegradation

**FIGURE 4:**
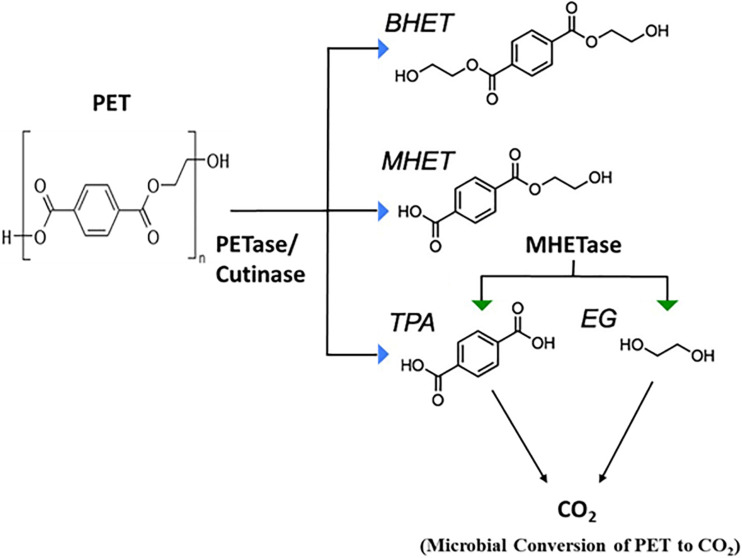
Microbial degradation of Polyethylene Terephthalate (PET) (adapted from Austin et al., 2018). *PETase*, polyethylene terephthalate (PET) hydrolase or PET-digesting enzyme; *BHET*, bis(2-hydroxyethyl) terephthalic acid; *MHET*, mono(2-hydroxyethyl) terephthalic acid; *TPA*, terephthalic acid; *EG*, Ethylene glycol.

**FIGURE 5:**
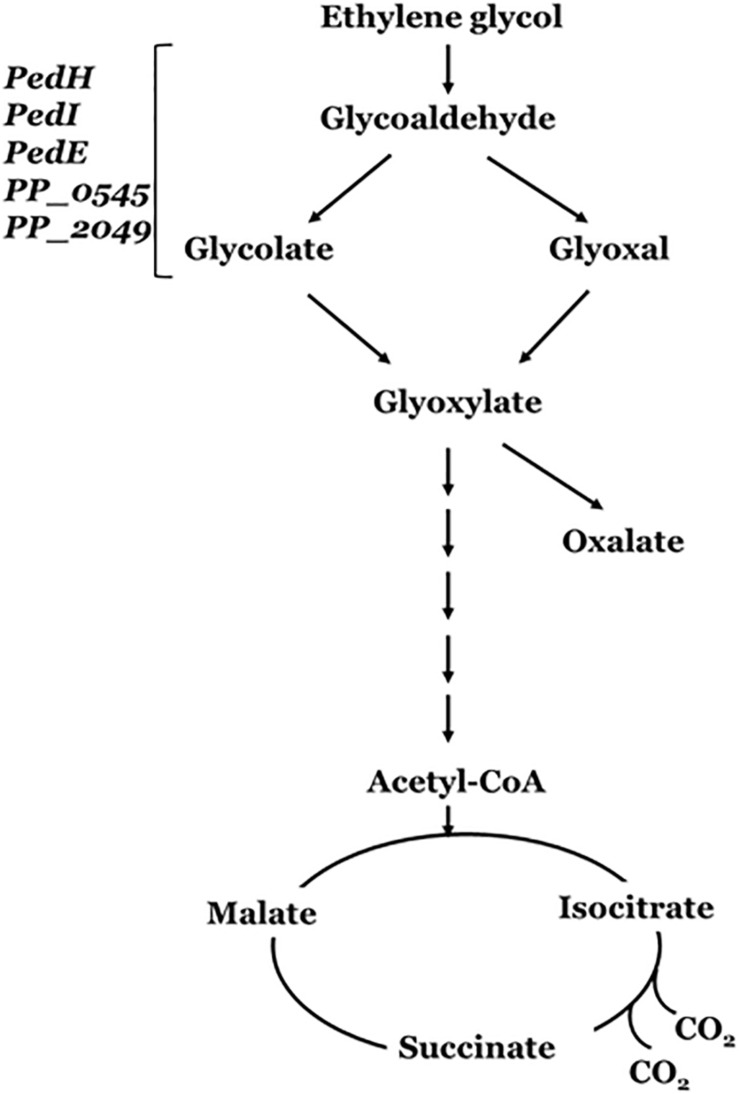
Ethylene glycol metabolism by *Pseudomonas putida*. PedE and PedH are the PQQ-dependent alcohol dehydrogenases (ADHs); PedI, PP_0545 and PP_2049 are the NADH-dependent aldehyde dehydrogenases (ALDHs). These enzyme encoding genes forms the phenylethanol degradation (Ped) cluster of *Pseudomonas putida* (adapted from Muckschel et al., 2012).

Yoshida et al. (2016) demonstrated that PET-digesting enzyme labeled as PETase, converts PET to mono(2-hydroxyethyl) terephthalic acid (MHET), with minimal amounts of terephthalic acid (TPA) and bis(2-hydroxyethyl)-TPA as secondary products (Figure 4). Another enzyme, MHETase (MHET-digesting enzyme), further hydrolyzes MHET into the two monomers, TPA and EG (Figure 4). Li et al. (2019) has reported the metabolism of EG, the second component of PET besides terephthalate, in *P. putida* KT2440. The metabolism of EG and its derivatives has resulted in different oxidation products such as glycolaldehyde, glyoxal, glycolate, and glyoxylate (Figure 5), which have a variety of value-added applications. These find use as reactive building blocks in the production of agro-, aroma-, and polymer chemicals, or pharmaceuticals (Sajtos, 1991; Mattioda and Christidis, 2000; Yue et al., 2012). Several microorganisms have been reported to utilize EG such as those from *Acetobacter* and *Gluconobacter* (DeLey and Kersters, 1964), *Acinetobacter* and halophilic bacterium, T-52 (ATCC 27042) (Gonzalez et al., 1972; Caskey and Taber, 1981), *Flavobacterium* species (Child and Willetts, 1978), *Hansenula* (Harada and Hirabayashi, 1968), *Candida*, *Pichia naganishii* AKU4267, and *Rhodotorula* sp. 3Pr-126 (Kataoka et al., 2001).

The genetic mechanism for the pathways enabling EG metabolism has been well demonstrated in *P. putida*. *P. putida* strain JM37 was able to utilize EG as a sole source of carbon and energy. However, while *P. putida* KT2440 was able to use EG a carbon source, it did not grow well. Both strains were able to metabolize EG and produced glycolic acid and glyoxylic acid (Figure 5). Initially, the diol is oxidized into glyoxylate in a series of reactions catalyzed by a set of dehydrogenases genes that encode the PP_0545, PedI, PedE, and PedH enzymes (Muckschel et al., 2012; Wehrmann et al., 2017). The conversion of EG to glyoxylate yields three reducing equivalents, either in the form of PQQH_2_, NADH, or in a direct coupling to the electron transport chain. Glyoxylate can be further metabolized by the Glyoxylate carboligase B (GclB) enzyme or AceA enzyme involved in the glyoxylate shunt (Blank et al., 2008) yielding two molecules of CO_2_ and two reducing equivalents.

In *P. putida* strain JM37, the activity of two additional pathways, namely, Gcl and GlcB, leads to rapid metabolism of EG without accumulation of the intermediates and/or oxalic acid (Muckschel et al., 2012). In case of *P. putida* U and *P. aeruginosa*, the Pyrroloquinoline quinone (PQQ)-dependent alcohol dehydrogenases involved are the PedE and PedH, and the ExaA (formerly QedH) enzymes (Arias et al., 2008). These periplasmic enzymes have been found essential for growth utilizing ethanol as a carbon source, since it catalyzes the oxidation of the substrate into acetaldehyde (Gorisch, 2003). Franden et al. (2018) has reported improved growth and EG utilization upon overexpression of Glyoxylate carboligase (*gcl)* operon in the engineered *P. putida* KT2440 (ATCC 47054) strain. In addition, the engineered strain enables conversion of EG to medium-chain-length polyhydroxyalkanoates (mcl-PHAs) (Mohanan et al., 2020).

## Petro-Plastic With Hetero/Carbamate(Urethane) Bonds: Polyurethane

Polyurethanes (PUs) are widely used synthetic polymers finding applications as microplastics in the medical (e.g., catheters) and industrial products (especially as foams and domestic consumables; Figure 1). PUs also finds use in adhesives, insulation, coats, tires, sponges, paints, and fibers (Howard, 2002; Zheng et al., 2005; Shah et al., 2008). PU is not abundant in nature and only a few microbial strains have been reported to efficiently degrade it. Extensive degradation of plastic waste generates microplastic (particles with a size smaller than 5 mm) (Zheng et al., 2005; Shah et al., 2008; do Sul and Costa, 2014). Microplastics pose a major problem to environment and human health, since the particles are shown to attract and store toxic compounds such as polybrominated diphenyl ethers (PBDs), polychlorinated biphenyls (PCBs) and bisphenols (Bouwmeester et al., 2015). Microplastics comprising PU foams, PE particles, and PP particles, form the major pollutants with a half-life estimated to be ∼50 years in sea water (Plastics Europe, 2017; Sharma and Chatterjee, 2017; Kim et al., 2015) and polluting coastal and marine habitats (Sharma and Chatterjee, 2017).

**FIGURE 6:**
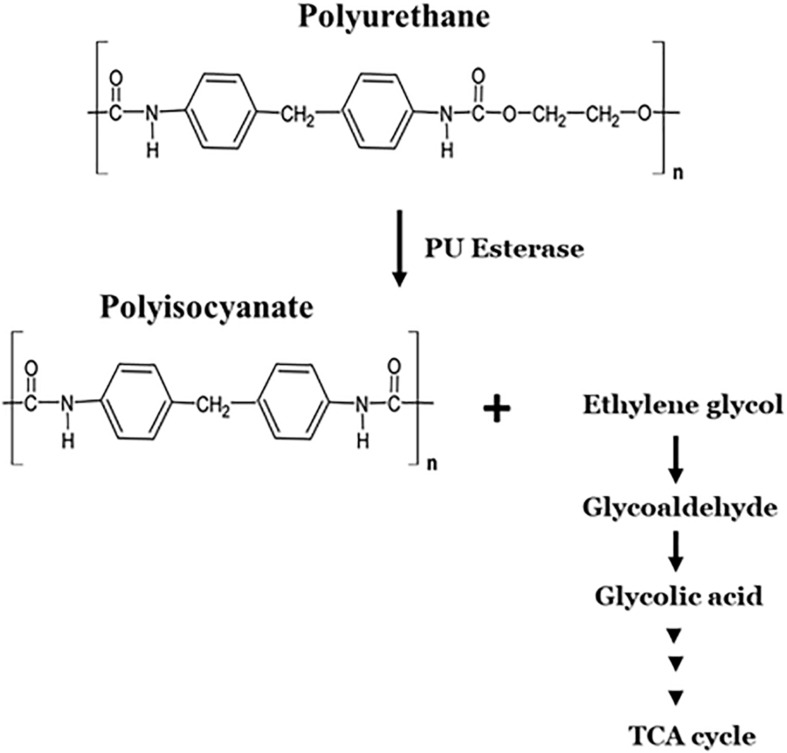
Microbial degradation pathway of polyurethane.

PU is biodegraded through hydrolytic cleavage of urethane bonds (Nakajima-Kambe et al., 1999; Figure 6). Few fungal and bacterial species are shown to degrade polyester-polyurethane through enzymatic hydrolysis of ester linkages (Nakajima-Kambe et al., 1999; Howard, 2002; Table 2). Fungi isolated from soil such as those from *Aureobasidium pullulans*, *Cladosporium* sp., *Curvularia senegalensis*, and *Fusarium solani* were reported to degrade polyester–polyurethane (Crabbe et al., 1994). A variety of bacterial strains were able to use polyester-polyurethane polymers as carbon, nitrogen and energy source for growth, e.g., *P. aeruginosa* (Kay et al., 1991), *Corynebacterium* sp. (Kay et al., 1991), *Comamonas acidovorans* (Nakajima-Kambe et al., 1995), *Pseudomonas fluorescens* (Howard and Blake, 1998), *Acinetobacter calcoaceticus* (El-Sayed et al., 1996), and *Bacillus subtilis* (Rowe and Howard, 2002).

## Enzymes Involved in Degradation of Synthetic Polymers

**FIGURE 7:**
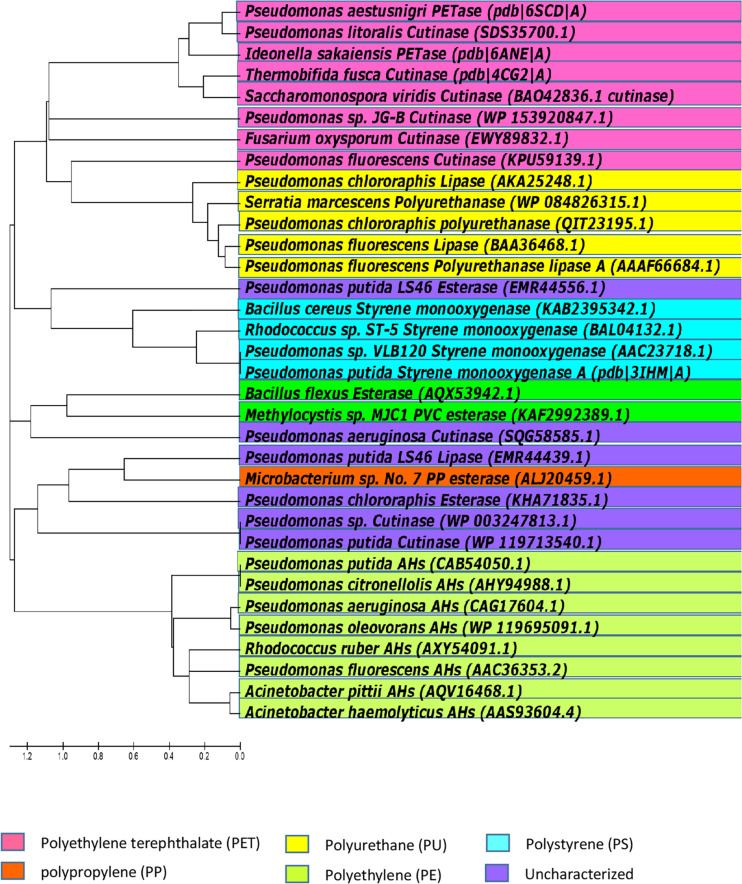
Phylogenetic relationships among bacteria, based on amino acid sequence homologies of petro-polymer degrading enzymes. Majority of the currently known and biochemically characterized, as well as uncharacterized enzymes, were included in the alignment. The tree was constructed with Molecular Evolutionary Genetics Analysis version 5 (MEGA5).

Petro-pastics are well known to be highly recalcitrant to natural biodegradation processes. However, as discussed above, some microorganisms can degrade certain polymers, under certain conditions. Microorganisms that degrade plastic may be considered living bioreactors. Polymer chains are broken by enzymes secreted by the microbes, and the hydrolysis products are taken up and metabolized. The identities of enzymes involved in the degradation petro-polymers like PE and PP, and the mechanisms of these enzyme-substrate interactions, are largely unknown. However, some progress in identifying the types of enzymes involve in petro-polymer degradation has been made. Enzymatic activities include hydrolysis/oxidation/hydroxylation, resulting in cleavage of the polymer chains into oligomers and monomers. From the point of view of enzymatic degradation, petro-polymers can be classified in two groups; hydrolysable (PET and PUR) and non-hydrolysable (PE, PS, PP, and PVC). The biodegradation pathways utilized by these two groups are significantly different (Wei and Zimmermann, 2017). The ester bonds in petro-plastic polymers are the same as ester bonds in other polyester polymers, and enzymes that cleave ester bonds are known as esterases. There are many different types of esterase, which differ in their protein structure, substrate specificity, and biological functions. Polymers with hydrolysable ester bonds in their backbones, such as PET and PUR, are more susceptible to biodegradation than polymers with carbon chain backbones, like PE, PS, PP and PVC. Enzymes that degrade the high-molecular weight polymers of PET and ester-based PU are known and details of their activities have been characterized. However, to date, no specific enzymes with the ability to biodegrade PE, PS, PP, or PVC have been well characterized, and the mechanisms of degradation the covalent bonds in carbon backbone petro-polymers are currently unknown. Figure 7 shows the selected enzymes from different microorganisms capable of hydrolyzing petro-polymers and synthetic polyesters.

It is presumed that large, high molecular weight polymers are first degraded by extracellular enzymes, secreted by microorganisms, into smaller subunits (oligomers and/or dimers) that can be incorporated into the microbial cells. Once in the cells, these degradation products are channelled through the classical catabolic pathways to yield energy and reducing power for cell growth. The microbial degradation of petro-polymers is known as a very slow process (Table 1). The high resistance of these polymers mainly stems from their high molecular weight and the extremely hydrophobic surfaces, which prevent these molecules from transiting the cell wall. Different levels of degradability are thought to depend on the extent of amorphous and crystalline forms, and the presence of strong C–C bonds, which are very resistant to attack by enzymes.

Cutinases (EC 3.1.1.74) are a sub-class of esterase enzymes that have gained importance because of their ability to hydrolyze polyesters with a high molar mass (Chen et al., 2013). Cutinases are α/β hydrolases or carboxylic ester hydrolases originally extracted from plant pathogenic fungi, e.g., *Fusarium solani pisi* (Purdy and Kolattukudy, 1975; Kollattukudy, 1981; Heredia, 2003). The enzyme caught attention for their phytopathogenicity as they can hydrolyze the cutin of the cuticular layer in leaves or the suberin in bark.

A polyesterase capable of hydrolyzing aromatic polyesters (primarily PET) was first reported from *Thermobifida fusca* by a German research group (Müller et al., 2005). A variety of cutinases were also purified and characterized from bacterial strains of thermophilic actinomycetes (Zimmermann and Billig, 2011; Chen et al., 2013; Wei et al., 2014a), for instance *Thermomonospora fusca* (Fett et al., 1999), *Thermobifida fusca* (Kleeberg et al., 2005), *Thermobifida alba* (Hu et al., 2010), *Thermobifida cellulosilytica* (Herrero Acero et al., 2011), and *Thermomonospora curvata* (Wei et al., 2014b; Islam et al., 2019). Cutinases have also been reported to hydrolyse aliphatic polyesters e.g., PCL (Kleeberg et al., 2005; Baker et al., 2012) as well as aliphatic aromatic co-polyesters such as PET (Herrero Acero et al., 2011; Ribitsch et al., 2012a; Roth et al., 2014; Wei et al., 2014b; Gamerith et al., 2017) and polytrimethylene terephthalate (PTT) (Eberl et al., 2009).

Several studies on PET degradation revealed that all known PET hydrolases belong to the cutinase group (Tokiwa and Calabia, 2007; Zimmermann and Billig, 2011; Baker et al., 2012). Further, crystal structures of PET-hydrolyzing cutinases have been analyzed for the substrate-binding domain and possible mutations to improve the degradation rates. Protein engineering studies on cutinases (Silva et al., 2011; Herrero Acero et al., 2013) and fusion to binding domains (Zhang et al., 2010; Ribitsch et al., 2013, 2015) have demonstrated enhanced polymer degradation. In latter approach, cutinases (from *T. cellulosilytica* and *T. fusca*) were fused to binding domains like polyhydroxyalkanoate binding modules (PBM) (Ribitsch et al., 2013), carbohydrate binding modules (CBM) (Zhang et al., 2010; Ribitsch et al., 2013) and hydrophobins (Ribitsch et al., 2015). In all cases, the activity and thus the hydrolysis toward PET was enhanced compared to the wild-type enzyme, which did not possess a hydrophobic binding-domain for a targeted polymer degradation. The fusion of hydrophobins (HBF4 or HBF7) to a PET-degrading cutinase (Thc_Cut1) enhanced the depolymerization up to 16 times (Ribitsch et al., 2015). Fusion of a PBM to the enzyme enhanced hydrolysis by 11-fold compared to the wild-type enzyme (Ribitsch et al., 2013).

Enzymatic hydrolysis of PET may occur via two mechanisms: (1) enzymatic surface modification of polyester fibers; or (2) enzymatic hydrolysis of the polymer. Enzymatic surface modification is carried out by PET surface-modifying enzymes or hydrolases, such as lipases, cutinases, carboxylesterases and proteases. These enzymes do not hydrolyze the PET polymer chains, but degrade the surface of the polymer. However, management of PET waste requires substantial degradation of the PET chains. Very few cutinases have been recognized as PET hydrolases that can degrade the inner block of PET (by at least 10%) since the first discovery of PET hydrolase by Müller et al. (2005).

Several hydrolases have been tested for the surface hydrophilization of PET fibers, such as lipases from *Candida antarctica* (Vertommen et al., 2005), *Triticum aestivum* (Nechwatal et al., 2006), *Thermomyces lanuginosus* (Eberl et al., 2009), and *Burkholderia* spp. (Lee and Chung, 2009); fungal cutinases from *Fusarium solani* (Alisch-Mark et al., 2006; O’Neill et al., 2007), *Penicillium citrinum* (Liebminger et al., 2007), and *Aspergillus oryzae*, *Humicola insolens* (Ronqvist et al., 2009); and those from actinomycetes, *Saccharomonospora viridis* (Kawai et al., 2017), *T. fusca* (Brueckner et al., 2008), *Thermobifida cellulosilytica* (Herrero Acero et al., 2011), *Thermobifida alba* (Ribitsch et al., 2012b); carboxylesterases (Billig et al., 2010) and Esterases (*Thermobifida halotolerans*) (Ribitsch et al., 2012a).

Besides crystallinity, hydrophobicity, surface topology, and molecular size of synthetic polymers (Tokiwa et al., 2009; Webb et al., 2013; Wei and Zimmermann, 2017), enzymatic PET degradation is also dependent on the reaction temperatures (>Tg, preferably 65–70°C in aqueous solution), and the enzyme structure (active site accessibility to the polymer surface; Zumstein et al., 2017; Islam et al., 2019). Polyesterases preferentially hydrolyzes amorphous regions of PET (Welzel et al., 2002; Brueckner et al., 2008; Ronqvist et al., 2009; Donelli et al., 2010; Gamerith et al., 2017). Increased crystallinity limits the movement of polymer chains and therefore, decreases the availability of polymer chains for enzymatic attack. None of the PET hydrolases reported so far degrades the crystalline polymers such as those in PET bottles, textiles, and biaxially stretched films. Degradation of the polyesters was found promoted at higher temperatures. Increased temperatures (over 55°C) also contributed to the disinfection of digested plastic waste (Islam et al., 2019).

Polyhydroxyalkanoate binding modules (PBM) have also been used to degrade polyurethane polyester co-polymers (Gamerith et al., 2016). The fusion of polyamidase and PBM (PA-PBM) improved hydrolysis of PU polymers by fourfold relative to the native enzyme (Gamerith et al., 2016). Many studies were conducted on the esterase-activities in the microbial degradation of polyester-polyurethane (Akutsu et al., 1998; Rowe and Howard, 2002). Membrane associated (e.g., PudA) (Akutsu et al., 1998) and extracellular (e.g., PueA, PueB) (Allen et al., 1999; Ruiz et al., 1999; Vega et al., 1999). Polyurethanases (PUases) from *Pseudomonas chlororaphis*, *P. fluorescens*, and *Comamonas acidovorans* TB-35) were characterized. The degradation of polyurethane, like other polymers, occurs in a two-step process with PU-esterase (PudA). Firstly, a surface-binding domain promotes hydrophobic adsorption of PudA to the PU surface and, hydrolysis of ester bonds forms the second step (Akutsu et al., 1998).

Islam et al. (2019) reported an improved binding and depolymerization of polyester-polyurethane nanoparticles by fusion of the anchor peptide, Tachystatin A2 (TA2) to the cutinase from *Thermomonospora curvata* (Tcur1278). Anchor peptides are metal-binding adhesion peptides, which provide a versatile tool for a targeted degradation of microplastics and/or polymers such as PET, PP, and PU, at ambient temperature in highly diluted suspensions. Tachystatin A2 (44 aa) has been known to from a dense monolayer on PP and PS surfaces (Osaki et al., 1999). TA2 was reengineered via a directed evolution campaign (following the KnowVolution strategy) (Rübsam et al., 2018b) and employing a specialized diversity generation protocol for short peptides (termed PePevo) to create variants with increased binding to PS and PP in the presence of surfactants (LAS and Triton X-100) (Rübsam et al., 2018a). The fusion of TA2 to the cutinase (Tcur1278) accelerated the degradation of polyester-polyurethane nanoparticles by a factor of 6.6 relative to the wild-type Tcur1278.

Blending of the plastics with certain types of natural polymers, such as starch, has been shown to increase their biodegradation (Otake et al., 1995; Zheng et al., 2005; Ammala et al., 2011; Karimi and Biria, 2019). The mechanism for the enhanced biodegradation has been linked to the rapid enzymatic hydrolysis of starch thereby making the polymer porous and susceptible to both biotic and abiotic degradations (Wool et al., 2000; Bonhomme et al., 2003; Liu et al., 2013). Karimi and Biria (2019) reported the biodegradation of LDPE-starch blend samples by an alpha-amylase aqueous solution. Amylase was found to have a co-metabolic behavior and hydrolyzed the primary specific substrate (starch) as well as PE molecules. Moreover, the addition of hydrophilic starch into hydrophobic PE was found to enhance the biodegradability of both polymers (Hoque et al., 2013).

The AHs of *Pseudomonas* sp. strain E4 were shown to play an important role in biodegradation of a non-oxidized LMWPE (Yoon et al., 2012). LMWPE has a molecular weight that was well above the upper-limit that can penetrate microbial membranes. Jeon and Kim (2016) reported the functional characteristics of alkane monooxygenases (AlkB1 and AlkB2) from *P. aeruginosa* involved in LMWPE biodegradation and found that the regulation mechanism of these enzymes was different, and that the AlkB2 enzyme was more effective in degrading LMWPE than the AlkB1 enzyme.

Danso et al. (2018) used Markov Model-based search strategy to show that a surprisingly large variety of potential polyesterases still needs to be discovered particularly in bacteria, which are currently not considered as a prime source for cutinases (Danso et al., 2018). The predominantly marine *Pseudomonas* lineage, which includes halophilic, psychrophilic, hydrocarbonoclastic, and heavy metal-tolerant species, is one such example; these were found as a source for PU hydrolyzing enzymes (Wilkes and Aristilde, 2017), while the Guebitz-group at the Austrian Centre of Industrial Biotechnology, Austria revealed the polyesterase activity in Pseudomonads (Haernvall et al., 2017; Wallace et al., 2017). Polyesterase genes in *Pseudomonas pertucinogena* have been confirmed by sequence homology searches (Bollinger et al., 2018). Further, many strains of *Pseudomonas* have been reported to degrade a wide-range of recalcitrant compounds and/or plastics such as PE, PS, PP, and PVC (Giacomucci et al., 2019).

## Conclusion

Biodegradation of petroleum-derived polymers has been an innovative area of research focused on solving plastic pollution in the environment. This review has discussed the microorganisms and enzymes reported to biodegrade these synthetic polymers. Many strains of *Pseudomonas* and *Bacillus* have been observed to degrade complex, recalcitrant compounds such as polyaromatic hydrocarbons, and have been associated with the partial degradation of a wide-range of petro-plastics, including PE, PS, PP, PVC, PET and ester-based PU. The gut microbes in insects have also been found to depolymerize PE, PS and PVC polymers. Enzymes specifically associated with depolymerization of PET and ester-based PU have been identified and intensively studied, while enzymes that effectively depolymerize PE, PP, PS, and PVC have not yet been identified and characterized. Further analyses of the genes and/or gene products (enzymes) that hydrolyze the high molecular weight petro-plastic polymers may lead to greater understanding of the underlying molecular mechanisms of biodegradation. Research focusing on digestive enzyme(s) in plastic-degrading invertebrates and their gut microbes could also lead to novel approach for plastic degradation, especially for persistent non-hydrolyzable polymers. Based on these knowledge, genetic engineering approaches to create recombinant microbial strains and/or enzymes could be adopted as the preferred strategy to enhance biodegradation of the synthetic petroleum based plastic waste.

## Author Contributions

NM was the primary author of the manuscript. ZM and DL contributed to certain sections of the manuscript, as well as overall editing and proof-reading. All authors contributed to the article and approved the submitted version.

## Conflict of Interest

The authors declare that the research was conducted in the absence of any commercial or financial relationships that could be construed as a potential conflict of interest.

## References

[B1] Abrusci C. Pablos J. L. Marín I. Espí E. Corrales T. Catalina F. (2013). Comparative effect of metal stearates as pro-oxidant additives on bacterial biodegradation of thermal- and photo-degraded low density polyethylene mulching films. *Int. Biodeterior. Biodegrad.* 83 25–32. 10.1016/j.ibiod.2013.04.002

[B2] Ahmed T. Shahid M. Azeem F. Rasul I. Shah A. A. Noman M. (2018). Biodegradation of plastics: current scenario and future prospects for environmental safety. *Environ. Sci. Pollut. Res. Int.* 25 7287–7298. 10.1007/s11356-018-1234-9 29332271

[B3] Akutsu Y. Nakajima-Kambe T. Nomura N. Nakahara T. (1998). Purification and properties of a polyester polyurethane-degrading enzyme from *Comamonas acidovorans* TB-35. *Appl. Environ. Microbiol.* 64 62–67. 10.1128/AEM.64.1.62-67.1998 16349494 PMC124672

[B4] Albertsson A. C. Anderson S. O. Karlsson S. (1987). Mechanism of biodegradation of polyethylene. *Polym. Degrad. Stab.* 18 73–87. 10.1016/0141-3910(87)90084-X

[B5] Albertsson A. C. Karlsson S. (1990). The influence of biotic and abiotic environments on the degradation of polyethylene. *Prog. Polym. Sci.* 15 177–192. 10.1016/0079-6700(90)90027-X

[B6] Albertsson A. C. Karlsson S. (1993). Aspects of biodeterioration of inert and degradable polymers. *Int. Biodeterior. Biodegrad.* 31 161–170. 10.1016/0964-8305(93)90002-J

[B7] Ali M. I. Ahmed S. Javed I. Ali N. Atiq N. Hameed A. (2014). Biodegradation of starch blended polyvinyl chloride films by isolated *Phanerochaete chrysosporium* PV1. *Int. J. Environ. Sci. Tech.* 11 339–348. 10.1007/s13762-013-0220-5

[B8] Alisch-Mark M. Herrmann A. Zimmermann W. (2006). Increase of the hydrophilicity of polyethylene terephthalate fibers by hydrolases from *Thermomonospora fusca* and *Fusarium solani* f. sp. pisi. *Biotechnol. Lett.* 28 681–685. 10.1007/s10529-006-9041-7 16791721

[B9] Allen A. B. Hilliard N. P. Howard G. T. (1999). Purification and characterization of a soluble polyurethane degrading enzyme from *Comamonas acidovorans*. *Int. Biodeterior. Biodegrad.* 43 37–41. 10.1016/S0964-8305(98)00066-3

[B10] Alvarez H. M. (2003). Relationship between β-oxidation pathway and the hydrocarbon-degrading profile in actinomycetes bacteria. *Int. Biodeterior. Biodegrad.* 52 35–42. 10.1016/S0964-8305(02)00120-8

[B234] Álvarez-Hernández C. Cairós C. López-Darias J. Mazzetti E. Hernández-Sánchez C. González-Sálamo J. (2019). Microplastic debris in beaches of Tenerife (Canary Islands, Spain). *Mar. Pollut. Bull.* 146, 26–32. 10.1016/j.marpolbul.2019.05.064 31426155

[B11] Ammala A. Bateman S. Deana K. Petinakis E. Sangwan P. Wong S. (2011). An overview of degradable and biodegradable polyolefins. *Prog. Polym. Sci.* 36 1015–1049. 10.1016/j.progpolymsci.2010.12.002

[B12] Andrady A. L. (2011). Microplastics in the marine environment. *Mar. Pollut. Bull.* 62 1596–1605. 10.1016/j.marpolbul.2011.05.030 21742351

[B13] Arias S. Olivera E. R. Arcos M. Naharro G. Luengo J. M. (2008). Genetic analyses and molecular characterization of the pathways involved in the conversion of 2-phenylethylamine and 2-phenylethanol into phenylacetic acid in *Pseudomonas putida* U. *Environ. Microbiol.* 10 413–432. 10.1111/j.1462-2920.2007.01464.x 18177365

[B14] Arkatkar A. Juwarkar A. A. Bhaduri S. Uppara P. V. Doble M. (2010). Growth of *Pseudomonas* and *Bacillus* biofilms on pretreated polypropylene surface. *Int. Biodeterior. Biodegrad.* 64 530–536. 10.1016/j.ibiod.2010.06.002

[B15] Austin H. P. Allen M. D. Donohoe B. S. Rorrer N. A. Kearns F. L. Silveira R. L. (2018). Characterization and engineering of a plastic degrading aromatic polyesterase. *Proc. Natl. Acad. Sci. U.S.A.* 115 E4350–E4357. 10.1073/pnas.1718804115 29666242 PMC5948967

[B16] Auta H. S. Emenike C. U. Fauziah S. H. (2017). Screening for Polypropylene degradation potential of bacteria isolated from mangrove ecosystems in Penninsular Malaysia. *IJBBB* 7 245–251. 10.17706/ijbbb.2017.7.4.245-251

[B17] Baker M. A.-M. Mead J. (2002). “Thermoplastics,” in *Handbook of Plastics, Elastomers and Composites*, 4th Edn, ed. Harper C. A. (New York, NY: McGraw-Hill), 1–90.

[B18] Baker P. J. Poultney C. Liu Z. Gross R. A. Montclare J. K. (2012). Identification and comparison of cutinases for synthetic polyester degradation. *Appl. Microbiol. Biotechnol.* 93 229–240. 10.1007/s00253-011-3402-4 21713515

[B19] Barnes D. K. A. Galgani F. Thompson R. C. Barlaz M. (2009). Accumulation and fragmentation of plastic debris in global environments. *Phil. Trans. R. Soc. B* 364 1985–1998. 10.1098/rstb.2008.0205 19528051 PMC2873009

[B20] Bergmann M. Mützel S. Primpke S. Tekman M. B. Trachsel J. Gerdts G. (2019). White and wonderful? Microplastics prevail in snow from the Alps to the Arctic. *Sci. Adv.* 5:eaax1157. 10.1126/sciadv.aax1157 31453336 PMC6693909

[B21] Bhatia S. K. Gurav R. Choi T. R. Jung H. R. Yang S. Y. Moon Y. M. (2019a). Bioconversion of plant biomass hydrolysate into bioplastic (polyhydroxyalkanoates) using *Ralstonia eutropha* 5119. *Bioresour. Technol.* 271 306–315. 10.1016/j.biortech.2018.09.122 30290323

[B22] Bhatia S. K. Gurav R. Choi T. R. Jung H. R. Yang S. Y. Song H. S. (2019b). Poly(3-hydroxybutyrate-*co*-3-hydroxyhexanoate) production from engineered *Ralstonia eutropha* using synthetic and anaerobically digested food waste derived volatile fatty acids. *Int. J. Biol. Macromol.* 133 1–10. 10.1016/j.ijbiomac.2019.04.083 30986452

[B23] Billen P. Khalifa L. Van Gerven F. Tavernier S. Spatari S. (2020). Technological application potential of polyethylene and polystyrene biodegradation by macro-organisms such as mealworms and wax moth larvae. *Sci. Total Environ.* 735:139521 10.1016/j.scitotenv.2020.139521 32470676

[B24] Billig S. Oeser T. Birkemeyer C. Zimmermann W. (2010). Hydrolysis of cyclic poly(ethylene terephthalate) trimers by a carboxylesterase from *Thermobifida fusca* KW3. *Appl. Microbiol. Biotechnol.* 87 1753–1764. 10.1007/s00253-010-2635-y 20467738

[B25] Blank L. M. Ionidis G. Ebert B. E. Bühler B. Schmid A. (2008). Metabolic response of *Pseudomonas putida* during redox biocatalysis in the presence of a second octanol phase. *FEBS J.* 275 5173–5190. 10.1111/j.1742-4658.2008.06648.x 18803670

[B26] Bollinger A. Thies S. Katzke N. Jaeger K. E. (2018). The biotechnological potential of marine bacteria in the novel lineage of *Pseudomonas pertucinogena*. *Microb. Biotechnol.* 13 19–31. 10.1111/1751-7915.13288 29943398 PMC6922532

[B27] Bombelli P. Howe C. J. Bertocchini F. (2017). Polyethylene bio-degradation by caterpillars of the wax moth *Galleria mellonella*. *Curr. Biol.* 27 292–293. 10.1016/j.cub.2017.02.060 28441558

[B28] Bonhomme S. Cuer A. Delort A. M. Lemaire J. Sancelme M. Scott G. (2003). Environmental biodegradation of polyethylene. *Polym. Degrad. Stab.* 81 441–452. 10.1016/S0141-3910(03)00129-0

[B29] Bouwmeester H. Hollman P. C. Peters R. J. (2015). Potential health impact of environmentally released micro-and nanoplastics in the human food production chain: experiences from nanotoxicology. *Environ. Sci. Technol.* 49 8932–8947. 10.1021/acs.est.5b01090 26130306

[B30] Brandon A. M. Gao S. H. Tian R. Ning D. Yang S. S. Zhou J. (2018). Biodegradation of polyethylene and plastic mixtures in mealworms (*Larvae of Tenebrio molitor*) and effects on the gut microbiome. *Environ. Sci.Technol.* 52 6526–6533. 10.1021/acs.est.8b02301 29763555

[B31] Braun D. (1975). “Recent progress in the thermal and photochemical degradation of poly(vinyl chloride),” in *Degradation and Stabilization of Polymers*, ed. Geuskens (London: Wiley), 23–41.

[B32] Braun D. (2004). Poly (vinyl chloride) on the way from the 19th century to the 21st century. *J. Polym. Sci. Part A Polym. Chem.* 42 578–586. 10.1002/pola.10906

[B33] Browne M. A. Crump P. Niven S. J. Teuten E. Tonkin A. Galloway T. (2011). Accumulation of microplastic on shorelines worldwide: sources and sinks. *Environ. Sci. Technol.* 45 9175–9179. 10.1021/es201811s 21894925

[B34] Brueckner T. Eberl A. Heumann S. Rabe M. Guebitz G. M. (2008). Enzymatic and chemical hydrolysis of poly(ethylene terephthalate) fabrics. *J. Polym. Sci. Pat A Polym. Chem.* 46 6435–6443. 10.1002/pola.22952

[B35] Bumpus J. A. Aust S. D. (1987). “Mineralization of recalcitrant environmental pollutants by the white rot fungus,” in *Proceedings of The National Conference on Hazardous Wastes and Hazardous Materials* Washington, DC, 146–151.

[B36] Bumpus J. A. Fernando T. Mileski G. J. Aust S. D. (1988). “Biological oxidation of organic compounds by enzymes from a white rot fungus,” in *Proceedings of The 14th Annual Research Symposium* Oak Ridge, TN, 1–16.

[B37] Cacciari P. Quatrini G. Zirletta E. Mincione V. Vinciguerra P. Lupattelli P. (1993). Isotactic polypropylene biodegradation by a microbial community: physicochemical characterization of metabolites produced. *Appl. Environ. Microbiol.* 59 3695–3700. 10.1128/AEM.59.11.3695-3700.1993 8285678 PMC182519

[B38] Carpenter E. J. Smith K. L. Jr. (1972). Plastics on the Sargasso Sea surface. *Science* 175 1240–1241. 10.1126/science.175.4027.1240 5061243

[B39] Caskey W. H. Taber W. A. (1981). Oxidation of ethylene glycol by a salt requiring bacterium. *Appl. Environ. Microbiol.* 42 180–183. 10.1128/AEM.42.1.180-183.1981 16345810 PMC243982

[B40] Cassone B. J. Grove H. C. Elebute O. Villanueva S. M. P. LeMoine C. M. R. (2020). Role of the intestinal microbiome in low-density polyethylene degradation by caterpillar larvae of the greater wax moth, Galleria mellonella. *Proc. R. Soc. B* 287:20200112 10.1098/rspb.2020.0112 PMC7126078 32126962

[B41] Chen S. Su L. Chen J. Wu J. (2013). Cutinase: characteristics, preparation and application. *Biotechnol. Adv.* 31 1754–1767. 10.1016/j.biotechadv.2013.09.005 24055682

[B42] Child J. Willetts A. (1978). Microbial metabolism of aliphatic glycols. Bacterial metabolism of ethylene glycol. *Biochim. Biophys. Acta* 538 316–327. 10.1016/0304-4165(78)90359-8 620072

[B43] Crabbe J. R. Campbell J. R. Thompson L. Walz S. L. Schultz W. W. (1994). Biodegradation of a colloidal ester-based polyurethane by soil fungi. *Int. Biodeterior. Biodegrad.* 33 103–113. 10.1016/0964-8305(94)90030-2

[B44] Danso D. Schmeisser C. Chow J. Zimmermann W. Wei R. Leggewie C. (2018). New insights into the function and global distribution of polyethylene terephthalate (PET)-degrading bacteria and enzymes in marine and terrestrial metagenomes. *Appl. Environ. Microbiol.* 84:e02773-17. 10.1128/AEM.02773-17 29427431 PMC5881046

[B45] Das G. Bordoloi N. K. Rai S. K. Mukherjee A. K. Karak N. (2012). Biodegradable and biocompatible epoxidized vegetable oil modified thermostable poly(vinyl chloride): thermal and performance characteristics post biodegradation with *Pseudomonas aeruginosa* and *Achromobacter* sp. *J. Hazard Mater.* 209-210 434–442. 10.1016/j.jhazmat.2012.01.043 22316688

[B46] Decker C. (1984). “Photodegratadion of PVC,” in *Degradation and Stabilization of PVC*, ed. Owen (London: Elsevier Applied Science Publication), 81–136. 10.1007/978-94-009-5618-6_3

[B47] DeLey J. Kersters K. (1964). Oxidation of aliphatic glycols by acetic acid bacteria. *Bacteriol. Rev.* 28 164–180. 10.1128/MMBR.28.2.164-180.1964 14172022 PMC441219

[B48] Di Gennaro P. Colmegna A. Galli E. Sello G. Pelizzoni F. Bestetti G. (1999). A new biocatalyst for production of optically pure aryl ep-oxides by styrene monooxygenase from *Pseudomonas fluorescens* ST. *Appl. Environ. Microbiol.* 65 2794–2797. 10.1128/AEM.65.6.2794-2797.1999 10347083 PMC91418

[B49] do Sul J. A. I. Costa M. F. (2014). The present and future of microplastic pollution in the marine environment. *Environ. Pollut.* 185 352–364. 10.1016/j.envpol.2013.10.036 24275078

[B50] Donelli I. Freddi G. Nierstrasz V. A. Taddei P. (2010). Surface structure and properties of poly(ethylene terephthalate) hydrolyzed by alkali and cutinase. *Polym. Degrad. Stab.* 95 1542–1550. 10.1016/j.polymdegradstab.2010.06.011

[B51] Eberl A. Heumann S. Brueckner T. Araujo R. Cavaco-Paulo A. Kaufmann F. (2009). Enzymatic surface hydrolysis of poly(ethylene terephthalate) and bis(benzoyloxyethyl)terephthalate by lipase and cutinase in the presence of surface active molecules. *J. Biotechnol.* 143 207–212. 10.1016/j.jbiotec.2009.07.008 19616594

[B52] El-Sayed A. H. M. M. Mahmoud W. M. Davis E. M. Coughlin R. W. (1996). Biodegradation of polyurethane coatings by hydrocarbon-degrading bacteria. *Int. Biodeterior. Biodegrad.* 37 69–79. 10.1016/0964-8305(95)00091-7

[B53] Eyheraguibel B. Traikia M. Fontanella S. Sancelme M. Bonhomme S. Fromageot D. (2017). Characterization of oxidized oligomers from polyethylene films by mass spectrometry and NMR spectroscopy before and after biodegradation by a *Rhodococcus rhodochrous* strain. *Chemosphere* 184 366–374. 10.1016/j.chemosphere.2017.05.137 28605707

[B54] Fett W. F. Wijey C. Moreau R. A. Osman S. F. (1999). Production of cutinase by *Thermomonospora fusca* ATCC 27730. *J. Appl. Microbiol.* 86 561–568. 10.1046/j.1365-2672.1999.00690.x

[B55] Fontanella S. Bonhomme S. Koutny M. Husarova L. Brusso J. M. Courdavault J. P. (2010). Comparison of the biodegradability of various polyethylene films containing pro-oxidant additives. *Polym. Degrad. Stab.* 95 1011–1021. 10.1016/j.polymdegradstab.2010.03.009

[B56] Franden M. A. Jayakody L. N. Li W.-J. Wagner N. J. Cleveland N. S. Michener W. E. (2018). Engineering *Pseudomonas putida* KT2440 for efficient ethylene glycol utilization. *Metab. Eng.* 48 197–207. 10.1016/j.ymben.2018.06.003 29885475

[B57] Gajendiran A. Krishnamoorthy S. Abraham J. (2016). Microbial degradation of low-density polyethylene (LDPE) by *Aspergillus clavatus* strain JASK1 isolated from landfill soil. *3 Biotech.* 6:52. 10.1007/s13205-016-0394-x 28330123 PMC4752946

[B58] Gamerith C. Herrero Acero E. Pellis A. Ortner A. Vielnascher R. Luschnig D. (2016). Improved enzymatic polyurethane hydrolysis by tuning enzymesorption. *Polym. Degrad. Stab.* 132 69–77. 10.1016/j.polymdegradstab.2016.02.025

[B59] Gamerith C. Zartl B. Pellis A. Guillamot F. Marty A. Herrero Acero E. (2017). Enzymatic recovery of polyester building blocks from polymer blends. *Process Biochem.* 59 58–64. 10.1016/j.procbio.2017.01.004

[B60] Geyer R. Jambeck J. R. Law K. L. (2017). Production, use, and fate of all plastics ever made. *Sci. Adv.* 3:e1700782. 10.1126/sciadv.1700782 28776036 PMC5517107

[B61] Giacomucci L. Raddadi N. Soccio M. Lotti N. Fava F. (2019). Polyvinyl chloride biodegradation by *Pseudomonas citronellolis* and *Bacillus flexus*. *New Biotechnol.* 52 35–41. 10.1016/j.nbt.2019.04.005 31026607

[B62] Gilan I. Hadar Y. Sivan A. (2004). Colonization, biofilm formation and biodegradation of polyethylene by a strain of *Rhodococcus ruber*. *Appl. Microbiol. Biotechnol.* 65 97–104. 10.1007/s00253-004-1584-8 15221232

[B63] Goldman A. S. (2010). ChemInform abstract: organometallic chemistry: carbon-carbon bonds get a break. *Nature* 463 435–436. 10.1038/463435a 20110980

[B64] Gonzalez C. F. Taber W. A. Zeitoun M. A. (1972). Biodegradation of ethylene glycol by a salt-requiring bacterium. *Appl. Microbiol.* 24 911–919. 10.1128/AEM.24.6.911-919.1972 4568254 PMC380695

[B65] Gorghiu L. M. Jipa S. Zaharescu T. Setnescu R. Mihalcea I. (2004). The effect of metals on thermal degradation of polyethylenes. *Polym. Degrad. Stab.* 84 7–11. 10.1016/S0141-3910(03)00265-9

[B66] Gorisch H. (2003). The ethanol oxidation system and its regulation in *Pseudomonas aeruginosa*. *Biochim. Biophys. Acta* 1647 98–102. 10.1016/S1570-9639(03)00066-9 12686116

[B67] Grima S. Bellon-Maurel V. Feuilloley P. Silvestre F. (2000). Aerobic biodegradation of polymers in solid-state conditions: a review of environmental and physicochemical parameter settings in laboratory simulations. *J. Polym. Environ.* 8 183–195. 10.1023/A:1015297727244

[B68] Guzik M. W. Kenny S. T. Duane G. F. Casey E. Woods T. Babu R. P. (2014). Conversion of postconsumer polyethylene to the biodegradable polymer polyhydroxyalkanoate. *Appl Microbiol Biotech.* 98 4223–4232. 10.1007/s00253-013-5489-2 24413975

[B69] Haernvall K. Zitzenbacher S. Wallig K. Yamamoto M. Schick M. B. Ribitsch D. (2017). Hydrolysis of ionic phthalic acid based polyesters by wastewater microorganisms and their enzymes. *Environ. Sci. Technol.* 51 4596–4605. 10.1021/acs.est.7b00062 28345898

[B70] Haines J. R. Alexander M. (1974). Microbial degradation of high-molecular-weight alkanes. *Appl. Microbiol.* 28 1084–1085. 10.1128/AEM.28.6.1084-1085.1974 4451369 PMC186890

[B71] Harada T. Hirabayashi T. (1968). Utilization of alcohols by Hansenula miso. *Agric. Biol. Chem.* 32 1175–1180. 10.1271/bbb1961.32.1175

[B72] Harrison J. P. Boardman C. O’Callaghan K. Delort A. M. Song J. (2018). Biodegradability standards for carrier bags and plastic films in aquatic environments: a critical review. *R. Soc. Open Sci.* 5:171792. 10.1098/rsos.171792 29892374 PMC5990801

[B73] Hasan F. Shah A. A. Hameed A. Ahmed S. (2007). Synergistic effect of photo and chemical treatment on the rate of biodegradation of low density polyethylene by *Fusarium* sp. AF4. *J. Appl. Polym. Sci.* 105 1466–1470. 10.1002/app.26328

[B74] Heredia A. (2003). Biochemical and biophysical characteristics of cutin, a plant barrier biopolymer. *Biochim. Biophys. Acta* 1620 1–7. 10.1016/S0304-4165(02)00510-X 12595066

[B75] Herrero Acero E. Ribitsch D. Steinkelner G. T. Gruber K. Greimel K. Eiteljoerg I. (2011). Enzymatic surface hydrolysis of PET: effect of structural diversity on kinetic properties of cutinases from *Thermobifida*. *Macromolecules* 44 4632–4640. 10.1021/ma200949p

[B76] Herrero Acero E. Ribitsch D. Dellacher A. Zitzenbacher S. Marold A. Steinkellner G. (2013). Surface engineering of a cutinase from Thermobifida cellulosilytica for improved polyester hydrolysis. *Biotechnol. Bioeng.* 110 2581–2590. 10.1002/bit.24930 23592055

[B77] Hidalgo-Ruz V. Gutow L. Thompson R. C. Thiel M. (2012). Microplastics in the marine environment: a review of the methods used for identification and quantification. *Environ. Sci. Technol.* 46 3060–3075. 10.1021/es2031505 22321064

[B78] Ho B. T. Roberts T. K. Lucas S. (2018). An overview on biodegradation of polystyrene and modified polystyrene: the microbial approach. *Crit. Rev. Biotechnol.* 38 1–13. 10.1080/07388551.2017.1355293 28764575

[B79] Hoque M. E. Ye T. J. Yong L. C. Mohd Dahlan K. Z. (2013). Sago starch-mixed low-density polyethylene biodegradable polymer: synthesis and characterization. *J. Mater.* 2013:365380 10.1155/2013/365380

[B80] Howard G. T. (2002). Biodegradation of polyurethane: a review. *Int. Biodeterior. Biodegrad.* 49 245–252. 10.1016/S0964-8305(02)00051-3

[B81] Howard G. T. Blake R. C. (1998). Growth of *Pseudomonas fluorescens* on a polyester-polyurethane and the purification and characterization of a polyurethanase-protease enzyme. *Int. Biodeterior. Biodegrad.* 42 213–220. 10.1016/S0964-8305(98)00051-1

[B82] Hu X. Thumarat U. Zhang X. Tang M. Kawai F. (2010). Diversity of polyester-degrading bacteria in compost and molecular analysis of a thermoactive esterase from *Thermobifida alba* AHK119. *Appl. Microbiol. Biotechnol.* 87 771–779. 10.1007/s00253-010-2555-x 20393707

[B83] Huang C.-Y. Roan M.-L. Kuo M.-C. Lu W.-L. (2005). Effect of compatibiliser on the biodegradation and mechanical properties of high-content starch/low-density polyethylene blends. *Polym. Degrad. Stab.* 90 95–105. 10.1016/j.polymdegradstab.2005.02.015

[B84] Islam S. Apitius L. Jakob F. Schwaneberg U. (2019). Targeting microplastic particles in the void of diluted suspensions. *Environ. Int.* 123 428–435. 10.1016/j.envint.2018.12.029 30622067

[B85] Iwamoto A. Tokiwa Y. (1994). Enzymatic degradation of plastics containing polycaprolactone. *Polym. Degrad. Stab.* 45 205–213. 10.1016/0141-3910(94)90138-4

[B86] Jain K. Bhunia H. Sudhakara Reddy M. (2018). Degradation of polypropylene-poly L lactide blend by bacteria isolated from compost. *Bioremed. J.* 22 3–4. 10.1080/10889868.2018.1516620

[B87] Jasso C. F. Gonzalez-Ortiz L. J. Contrews J. R. Mendizabal M. E. Mora G. J. (1998). The degradation of high impact polystyrene with and without starch in concentrated activated sludge. *Polym. Eng. Sci.* 38 863–869. 10.1002/pen.10252

[B88] Jeon H. J. Kim M. N. (2015). Functional analysis of alkane hydroxylase system derived from *Pseudomonas aeruginosa* E7 for low molecular weight polyethylene biodegradation. *Int. Biodeterior. Biodegrad.* 103 141–146. 10.1016/j.ibiod.2015.04.024

[B89] Jeon H. J. Kim M. N. (2016). Comparison of the functional characterization between alkane monooxygenases for low-molecular-weight polyethylene biodegradation. *Int. Biodeterior. Biodegrad.* 114 202–208. 10.1016/j.ibiod.2016.06.012

[B90] Jumaah O. S. (2017). Screening of plastic degrading bacteria from dumped soil area. *IOSR J. Environ. Sci. Toxicol. Food Technol.* 11 93–98. 10.9790/2402-1105029398

[B91] Kaczmarek H. Bajer K. (2007). Biodegradation of plasticized poly(vinyl chloride) containing cellulose. *J. Polym. Sci. Part B Polym. Phys.* 45 903–918. 10.1002/polb.21100

[B92] Kaczmarek H. Oldak D. Malanowski P. Chaberska H. (2005). Effect of short wavelength UV-irradiation on ageing of polypropylene/cellulose compositions. *Polym. Degrad. Stab.* 88 189–198. 10.1016/j.polymdegradstab.2004.04.017

[B93] Kaplan D. L. Roy H. Jim S. (1979). Biodegradation of polystyrene, poly(methyl methacrylate), and phenol formaldehyde. *Appl. Environ. Microbiol.* 38 551–553. 10.1128/AEM.38.3.551-553.1979 533278 PMC243531

[B94] Karimi M. Biria D. (2019). The promiscuous activity of alpha-amylase in biodegradation of low density polyethylene in a polymer-starch blend. *Nat. Sci. Rep.* 9:2612. 10.1038/s41598-019-39366-0 30796314 PMC6385501

[B95] Kataoka M. Sasaki M. Hidalgo A. R. Nakano M. Shimizu S. (2001). Glycolic acid production using ethylene glycol-oxidizing microorganisms. *Biosci. Biotechnol. Biochem.* 65 2265–2270. 10.1271/bbb.65.2265 11758919

[B96] Kawai F. Kawase T. Shiono T. Urakawa H. Sukigara S. Tu C. (2017). Enzymatic hydrophilization of polyester fabrics using a recombinant cutinase Cut190 and their surface characterization. *J. Fiber Sci. Technol.* 73 8–18. 10.2115/fiberst.fiberst.2017-0002

[B97] Kawai F. Oda M. Tamashiro T. Waku T. Tanaka N. Yamamoto M. (2014). A novel Ca2+-activated, thermostabilized polyesterase capable of hydrolyzing polyethylene terephthalate from *Saccharomonospora viridis* AHK 190. *Appl. Microbiol. Biotechnol.* 98 10053–10064. 10.1007/s00253-014-5860-y 24929560

[B98] Kay M. J. Morton L. H. G. Prince E. L. (1991). Bacterial degradation of polyester polyurethane. *Int. Biodeterior. Biodegrad.* 27 205–222. 10.1016/0265-3036(91)90012-G

[B99] Kenny S. T. Runic J. N. Kaminsky W. Woods T. Babu R. P. Keely C. M. (2008). Up-cycling of PET (polyethylene terephthalate) to the biodegradable plastic PHA (polyhydroxyalkanoate). *Environ. Sci. Technol.* 42 7696–7701. 10.1021/es801010e 18983095

[B100] Kenny S. T. Runic J. N. Kaminsky W. Woods T. Babu R. P. O’Connor K. E. (2012). Development of a bioprocess to convert PET derived terephthalic acid and biodiesel derived glycerol to medium chain length polyhydroxyalkanoate. *Appl. Microbiol. Biotechnol.* 95 623–633. 10.1007/s00253-012-4058-4 22581066

[B101] Kijchavengkul T. Auras R. (2008). Compostability of polymers. *Polym. Int.* 57 793–804. 10.1002/pi.2420

[B102] Kim H. R. Lee H. M. Yu H. C. Jeon E. Lee S. Li J. J. (2020). Biodegradation of polystyrene by *Pseudomonas* sp. isolated from the gut of superworms (Larvae of *Zophobas atratus*). *Environ. Sci. Technol.* 54 6987–6996. 10.1021/acs.est.0c01495 32374590

[B103] Kim M. Hyun S. Kwon J.-H. (2015). Estimation of the environmental load of high- and low-density polyethylene from South Korea using a mass balance approach. *Arch. Environ. Contam. Toxicol.* 69 367–373. 10.1007/s00244-015-0192-1 26153107

[B104] Kleeberg I. Hetz C. Kroppenstedt R. M. Müller R.-J. Deckwer W.-D. (1998). Biodegradation of aliphatic-aromatic copolyesters by *Thermomonospora fusca* and other thermophilic compost isolates. *Appl. Environ. Microbiol.* 64 1731–1735. 10.1128/AEM.64.5.1731-1735.1998 9572944 PMC106223

[B105] Kleeberg I. Welzel K. Vandenheuvel J. Müller R.-J. Deckwer W.-D. (2005). Characterization of a new extracellular hydrolase from *Thermobifida fusca* degrading aliphatic-aromatic copolyesters. *Biomacromolecules* 6 262–270. 10.1021/bm049582t 15638529

[B106] Klrbas Z. Keskin N. Güner A. (1999). Biodegradation of polyvinylchloride (PVC) by white rot fungi. *Bull. Environ. Contam. Toxicol.* 63 335–342. 10.1007/s001289900985 10475911

[B107] Kollattukudy P. E. (1981). Structure, biosynthesis, and biodegradation of cutin and suberin. *Annu. Rev. Plant Physiol.* 32 539–567. 10.1146/annurev.pp.32.060181.002543

[B108] Kundungal H. Gangarapu M. Sarangapani S. Patchaiyappan A. Devipriya S. P. (2019). Efficient biodegradation of polyethylene (HDPE) waste by the plastic-eating lesser waxworm (*Achroia grisella*). *Environ. Sci. Pollut. Res. Int.* 26 18509–18519. 10.1007/s11356-019-05038-9 31049864

[B109] Kyaw B. M. Champakalakshmi R. Sakharkar M. K. Lim C. S. Sakharkar K. R. (2012). Biodegradation of low density polythene (LDPE) by *Pseudomonas* species. *Indian J. Microbiol.* 52 411–419. 10.1007/s12088-012-0250-6 23997333 PMC3460136

[B110] Lee C. W. Chung J. D. (2009). Synthesis and biodegradation behavior of poly(ethylene terephthalate) oligomers. *Polymer Korea* 33 198–202.

[B111] Li W.-J. Jayakody L. N. Franden M. A. Wehrmann M. Daun T. Hauer B. (2019). Laboratory evolution reveals the metabolic and regulatory basis of ethylene glycol metabolism by *Pseudomonas putida* KT2440. *Environ. Microbiol.* 21 3669–3682. 10.1111/1462-2920.14703 31166064

[B112] Liebminger S. Eberl A. Sousa F. Heumann S. Fischer-Colbrie G. Cavaco-Paulo A. (2007). Hydrolysis of PET and bis-(benzoyloxyethyl) terephthalate with a new polyesterase from *Penicillium citrinum*. *Biocatal. Biotransform.* 25 171–177. 10.1080/10242420701379734

[B113] Liu X. Yu L. Xie F. Petinakis E. Sangwan P. Shen S. (2013). New evidences of accelerating degradation of polyethylene by starch. *J. Appl. Polym. Sci.* 130 2282–2287. 10.1002/app.39421

[B114] Lou Y. Ekaterina P. Yang S. S. Lu B. Liu B. Ren N. (2020). Biodegradation of polyethylene and polystyrene by greater wax moth larvae (*Galleria mellonella* L.) and the effect of co-diet supplementation on the core gut microbiome. *Environ. Sci. Technol.* 54 2821–2831. 10.1021/acs.est.9b07044 32013402

[B115] Marques-Calvo M. S. Cerda-Cuellar M. Kint D. P. R. Bou J. J. Munoz-Guerra S. (2006). Enzymatic and microbial biodegradability of poly(ethylene terephthalate) copolymers containing nitrated units. *Polym. Degrad. Stab.* 91 663–671. 10.1016/j.polymdegradstab.2005.05.014

[B116] Matthews S. Belcher J. D. Tee K. L. Girvan H. M. Mclean K. J. Rigby S. E. (2017). Catalytic determinants of alkene production by the cytochrome P450 peroxygenase OleTJE. *J. Biol. Chem.* 292 5128–5143. 10.1074/jbc.M116.762336 28053093 PMC5377825

[B117] Mattioda G. Christidis Y. (2000). “Glyoxylic acid,” in *Ullmann’s Encyclopedia of Industrial Chemistry*, eds Bohnet M. Brinker C. G. Cornils B. (Weinheim: Wiley-VCH Verlag GmbH & Co KGaA), 89–92.

[B118] Mohan A. J. Sekhar V. C. Bhaskar T. Nampoothiri K. M. (2016). Microbial assisted high impact polystyrene (HIP) degradation. *Bioresour. Technol.* 213 204–207. 10.1016/j.biortech.2016.03.021 26993201

[B119] Mohanan N. Sharma P. K. Levin D. B. (2020). Characterization of an intracellular poly(3-hydroxyalkanoate) depolymerase from the soil bacterium, *Pseudomonas putida* LS46. *Polym. Degrad. Stab.* 175:109127 10.1016/j.polymdegradstab.2020.109127

[B120] Montazer Z. Habibi Najafi M. B. Levin D. B. (2019). Microbial degradation of low-density polyethylene and synthesis of polyhydroxyalkanoate polymers. *Can. J. Microbiol.* 65 1–11. 10.1139/cjm-2018-0335 30485122

[B121] Montazer Z. Habibi Najafi M. B. Levin D. B. (2020a). Challenges with verifying microbial degradation of polyethylene. *Polymers* 12:123. 10.3390/polym12010123 31948075 PMC7022683

[B122] Montazer Z. Habibi Najafi M. B. Levin D. B. (2020b). In vitro degradation of low-density polyethylene by new bacteria from larvae of the Greater Wax Moth, *Galleria melonella*. *Can. J. Microbiol.* 10.1139/cjm-2020-0208 33306436

[B123] Mor R. Sivan A. (2008). Biofilm formation and partial biodegradation of polystyrene by the actinomycete *Rhodococcus ruber*: biodegradation of polystyrene. *Biodegradation* 19 851–858. 10.1007/s10532-008-9188-0 18401686

[B124] Morancho J. M. Ramis X. Fernandez X. Cadenato A. Salla J. M. (2006). Calorimetric and thermogravimetric studies of UV irradiated polypropylene/starch-based materials aged in soil. *Polym. Degrad. Stab.* 91 44–51. 10.1016/j.polymdegradstab.2005.04.029

[B125] Motta O. Proto A. De Carlo F. De Caro F. Santoro E. Brunetti L. (2009). Utilization of chemically oxidized polystyrene as co-substrate by filamentous fungi. *Int. J. Hygiene Environ. Health* 212 61–66. 10.1016/j.ijheh.2007.09.014 18222723

[B126] Muckschel B. Simon O. Klebensberger J. Graf N. Rosche B. Altenbuchner J. (2012). Ethylene glycol metabolism by *Pseudomonas putida*. *Appl. Environ. Microbiol.* 78 8531–8539. 10.1128/AEM.02062-12 23023748 PMC3502918

[B127] Muller R.-J. Kleeberg I. Deckwer W.-D. (2001). Biodegradation of polyesters containing aromatic constituents. *J. Biotechnol.* 86 87–95. 10.1016/S0168-1656(00)00407-7 11245897

[B128] Müller R.-J. Schrader H. Profe J. Dresler K. Deckwer W.-D. (2005). Enzymatic degradation of poly(ethylene terephthalate): rapid hydrolysis using a hydrolase from *T. fusca*. *Macromol. Rapid Commun.* 26 1400–1405. 10.1002/marc.200500410

[B129] Nakajima-Kambe T. Onuma F. Kimpara N. Nakahara T. (1995). Isolation and characterization of a bacterium which utilizes polyester polyurethane as a sole carbon and nitrogen source. *FEMS Microbiol. Lett.* 129 39–42. 10.1111/j.1574-6968.1995.tb07554.x 7781989

[B130] Nakajima-Kambe T. Shigeno-Akutsu Y. Nomura N. Onuma F. Nakahara T. (1999). Microbial degradation of polyurethane, polyester polyurethanes and polyether polyurethanes. *Appl. Microbiol. Biotechnol.* 51 134–140. 10.1007/s002530051373 10091317

[B131] Nakamiya K. Sakasita G. Ooi T. Kinoshita S. (1997). Enzymatic degradation of polystyrene by hydroquinone peroxidase of *Azotobacter beijerinckii* HM121. *J. Biosci. Bioeng.* 84 480–482. 10.1016/S0922-338X(97)82013-2

[B132] Nechwatal A. Blokesch A. Nicolai M. Krieg M. Kolbe A. Wolf M. (2006). A contribution to the investigation of enzyme catalysed hydrolysis of poly(ethylene terephthalate) oligomers. *Macromol. Mater. Eng.* 291 1486–1494. 10.1002/mame.200600204

[B133] Neufeld L. Stassen F. Sheppard R. Gilman T. (2016). *The New Plastics Economy: Rethinking the Future of Plastics.* Cologny: World Economic Forum.

[B134] Nikolic V. Sava V. Dusan A. Aleksander P. (2013). Biodegradation of starch-graft-polystyrene and starch-graft-poly(methacrylic acid) copolymers in model river water. *J. Serb. Chem. Soc.* 78 1425–1441. 10.2298/JSC121216051N

[B135] Nowak B. Paja̧ K. J. Drozd-Bratkowicz M. Rymarz G. (2011). Microorganisms participating in the biodegradation of modified polyethylene films in different soils under laboratory conditions. *Int. Biodeterior. Biodegrad.* 65 757–767. 10.1016/j.ibiod.2011.04.007

[B136] Oda M. Yamagami Y. Inaba S. Oida T. Yamamoto M. Kitajima S. (2018). Enzymatic hydrolysis of PET: functional roles of three Ca2+ ions bound to a cutinase-like enzyme, Cut190^∗^, and its engineering for improved activity. *Appl. Microbiol. Biotechnol.* 102 10067–10077. 10.1007/s00253-018-9374-x 30250976

[B137] Odusanya S. A. Nkwogu J. V. Alu N. Etuk Udo G. A. Ajao J. A. Osinkolu G. A. (2013). Preliminary studies on microbial degradation of plastics used in packaging potable water in Nigeria. *Niger. Food J.* 31 63–72. 10.1016/S0189-7241(15)30078-3

[B138] Ojeda T. (2013). “Polymers and the environment,” in *Polymer Science*, ed. Yılmaz F. (Rijeka: InTech), 1–34. 10.5772/51057

[B139] Ojeda T. Freitas A. Birck K. Dalmolin E. Jacques R. Bento F. (2011). Degradability of linear polyolefins under natural weathering. *Polym. Degrad. Stab.* 96 703–707. 10.1016/j.polymdegradstab.2010.12.004

[B140] Ojeda T. Freitas A. Dalmolin E. Pizzol M. D. Vignol L. Melnik J. (2009). Abiotic and biotic degradation of oxo-biodegradable foamed polystyrene. *Polym. Degrad. Stab.* 94 2128–2133. 10.1016/j.polymdegradstab.2009.09.012

[B141] O’Neill A. Araújo R. Casal M. Guebitz G. Cavaco-Paulo A. (2007). Effect of the agitation on the adsorption and hydrolytic efficiency of cutinases on polyethylene terephthalate fibres. *Enzym. Microb. Technol.* 40 1801–1805. 10.1016/j.enzmictec.2007.02.012

[B142] Osaki T. Omotezako M. Nagayama R. Hirata M. Iwanaga S. Kasahara J. (1999). Horseshoe crab hemocyte-derived antimicrobial polypeptides, tachystatins, with sequence similarity to spider neurotoxins. *J. Biol. Chem.* 274 26172–26178. 10.1074/jbc.274.37.26172 10473569

[B143] Otake Y. Kobayashi T. Asabe H. Murakami N. Ono K. (1995). Biodegradation of low density polyethylene, polystyrene, polyvinyl chloride, and urea formaldehyde resin buried under soil for over 32 years. *J. Appl. Polym. Sci.* 56 1789–1796. 10.1002/app.1995.070561309

[B144] Owen E. D. (1976). Photodegradation of polyvinyl chloride. *ACS Symp. Ser.* 25 208–219. 10.1021/bk-1976-0025.ch015

[B145] Owen J. (2012). *Degradation and Stabilisation of PVC.* New York, NY: Springer Science & Business Media.

[B146] Owen S. Otani T. Masaoka S. Ohe T. (1996). The biodegradation of low-molecular weight urethane compounds by a strain of *Exophiala jeanselmei*. *Biosci. Biotechnol. Biochem.* 60 244–248. 10.1271/bbb.60.244 27299400

[B147] Peixoto J. Silva L. P. Krüger R. H. (2017). Brazilian Cerrado soil reveals an untapped microbial potential for unpretreated polyethylene biodegradation. *J. Hazard. Mater.* 324 634–644. 10.1016/j.jhazmat.2016.11.037 27889181

[B148] Peng B. Y. Chen Z. Chen J. Yu H. Zhou X. Criddle C. S. (2020a). Biodegradation of polyvinyl chloride (PVC) in *Tenebrio molitor* (Coleoptera Tenebrionidae) larvae. *Environ. Int.* 145:106106. 10.1016/j.envint.2020.106106 32947161

[B149] Peng B. Y. Li Y. Fan R. Chen Z. Chen J. Brandon A. M. (2020b). Biodegradation of low-density polyethylene and polystyrene in superworms, larvae of *Zophobas atratus* (Coleoptera: Tenebrionidae): broad and limited extent depolymerization. *Environ. Poll.* 266:115206. 10.1016/j.envpol.2020.115206 32682160

[B150] Peng B. Y. Su Y. Chen Z. Chen J. Zhou X. Benbow M. E. (2019). Biodegradation of polystyrene by dark (*Tenebrio obscurus*) and yellow (*Tenebrio molitor*) mealworms (*Coleoptera: Tenebrionidae*). *Environ. Sci. technol.* 53 5256–5265. 10.1021/acs.est.8b06963 30990998

[B151] Pirt S. J. (1980). Microbial degradation of synthetic polymers. *J. Chem. Technol. Biotechnol.* 30 176–179. 10.1002/jctb.503300122

[B152] Plastics Additives (1998). *Plastics Additives*, ed. Pritchard G. (Dordrecht: Springer).

[B153] Plastics Europe. (2017). *Plastics – The Facts 2017: An Analysis of European Plastics Production, Demand and Waste Data 2017.* Brussels: Plastics Europe.

[B154] Purdy R. E. Kolattukudy P. E. (1975). Hydrolysis of plant cuticle by plant pathogens. Properties of cutinase I, cutinase II, and a nonspecific esterase isolated from *Fusarium solani pisi*. *Biochemistry* 14 2832–2840. 10.1021/bi00684a007 239740

[B155] Pushpadass H. A. Weber R. W. Dumais J. J. Hanna M. A. (2010). Biodegradation characteristics of starch-polystyrene loose-fill foams in a composting medium. *Bioresour. Technol.* 101 7258–7264. 10.1016/j.biortech.2010.04.039 20472424

[B156] Ragaert K. Delva L. Van Geem K. (2017). Mechanical and chemical recycling of solid plastic waste. *Waste Manage.* 69 24–58. 10.1016/j.wasman.2017.07.044 28823699

[B157] Rajandas H. Parimannan S. Sathasivam K. Ravichandran M. Yin L. S. (2012). A novel FTIR-ATR spectroscopy based technique for the estimation of low-density polyethylene biodegradation. *Polym. Test.* 31 1094–1099. 10.1016/j.polymertesting

[B158] Ramis X. Cadenato A. Salla J. M. Morancho J. M. Valles A. Contat L. (2004). Thermal degradation of polypropylene/starch based materials with enhanced biodegradability. *Polym. Degrad. Stab.* 86 483–491. 10.1016/j.polymdegradstab.2004.05.021

[B159] Ren L. Men L. Zhang Z. Guan F. Tian J. Wang (2019). Biodegradation of polyethylene by *Enterobacter* sp. D1 from the guts of Wax Moth *Galleria mellonella*. *Int. J. Environ. Res. Publ. Health* 16:1941. 10.3390/ijerph16111941 31159351 PMC6604253

[B160] Restrepo-Flórez J.-M. Bassi A. Thompson M. R. (2014). Microbial degradation and deterioration of polyethylene. A review. *Int. Biodeterior. Biodegrad.* 88 83–90. 10.1016/j.ibiod.2013.12.014

[B161] Ribitsch D. Herrero Acero E. Greimel K. Dellacher A. Zitzenbacher S. Marold A. (2012a). A new esterase from *Thermobifida halotolerans* hydrolyses polyethylene terephthalate (PET) and polylactic acid (PLA). *Polymers* 4 617–629. 10.3390/polym4010617

[B162] Ribitsch D. Herrero Acero E. Greimel K. Eiteljoerg I. Trotscha E. Freddi G. (2012b). Characterization of a new cutinase from *Thermobifida alba* for PET-surface hydrolysis. *Biocatal. Biotransform.* 30 2–9. 10.3109/10242422.2012.644435

[B163] Ribitsch D. Herrero Acero E. Przylucka A. Zitzenbacher S. Marold A. Schwab H. (2015). Enhanced cutinase-catalyzed hydrolysis of polyethylene terephthalate by covalent fusion to hydrophobins. *Appl. Environ. Microbiol.* 81 3586–3592. 10.1128/AEM.04111-14 25795674 PMC4421044

[B164] Ribitsch D. Heumann S. Trotscha E. Acero E. H. Greimel K. Leber R. (2011). Hydrolysis of polyethyleneterephthalate by *p*-nitrobenzylesterase from *Bacillus subtilis*. *Biotechnol. Progress* 27 951–960. 10.1002/btpr.610 21574267

[B165] Ribitsch D. Yebra A. O. Zitzenbacher S. Wu J. Nowitsch S. Steinkellner G. (2013). Fusion of binding domains to *Thermobifida cellulosilytica* cutinase to tune sorption characteristics and enhancing PET hydrolysis. *Biomacromolecules* 14 1769–1776. 10.1021/bm400140u 23718548

[B166] Riudavets J. Salas I. Pons M. J. (2007). Damage characteristics produced by insect pests in packaging film. *J. Stored Prod. Res.* 43 564–570. 10.1016/j.jspr.2007.03.006

[B167] Rojo F. (2009). Degradation of alkanes by bacteria. *Environ. Microbiol.* 11 2477–2490. 10.1111/j.1462-2920.2009.01948.x 19807712

[B168] Ronqvist ÅM. Xie W. Lu W. Gross R. A. (2009). Cutinase-catalyzed hydrolysis of poly(ethylene terephthalate). *Macromolecules* 42 5128–5138. 10.1021/ma9005318

[B169] Roth C. Wei R. Oeser T. Then J. Foellner C. Zimmermann W. (2014). Structural and functional studies on a thermostable polyethylene terephthalate degrading hydrolase from *Thermobifida fusca*. *Appl. Microbiol. Biotechnol.* 98 7815–7823. 10.1007/s00253-014-5672-0 24728714

[B170] Rowe L. Howard G. T. (2002). Growth of *Bacillus subtilis* on polyurethane and the purification and characterization of a polyurethanase-lipase enzyme. *Int. Biodeterior. Biodegrad.* 50 33–40. 10.1016/S0964-8305(02)00047-1

[B171] Rübsam K. Davari M. D. Jakob F. Schwaneberg U. (2018a). KnowVolution of the polymer-binding peptide LCI for improved polypropylene binding. *Polymers* 10:423. 10.3390/polym10040423 30966458 PMC6415234

[B172] Rübsam K. Weber L. Jakob F. Schwaneberg U. (2018b). Directed evolution of polypropylene and polystyrene binding peptides. *Biotechnol. Bioeng.* 115 321–330. 10.1002/bit.26481 29064564

[B173] Ruiz C. Main T. Hilliard N. P. Howard G. T. (1999). Purification and characterization of twopolyurethanase enzymes from *Pseudomonas chlororaphis*. *Int. Biodeterior. Biodegrad.* 43 43–47. S0964-8305(98)00067-5 10.1016/S0964-8305(98)00067-5

[B174] Sahebnazar Z. Shojaosadati S. A. Mohammad-Taheri M. Nosrati M. (2010). Biodegradation of low-density polyethylene (LDPE) by isolated fungi in solid waste medium. *Waste Manage.* 30 396–401. 10.1016/j.wasman.2009.09.027 19919893

[B175] Sajtos A. (1991). *Process for the Preparation of Glyoxylic Acid and Glyoxylic Acid Derivates. US Patent No 5,015,760.*

[B176] Sameh A. S. Alariqi, Pradeep Kumar A. Rao B. S. M. Singh R. P. (2006). Biodegradation of γ-sterilized biomedical polyolefins under composting and fungal culture environments. *Polym. Degrad. Stab.* 91 1105–1116. 10.1016/j.polymdegradstab.2005.07.004

[B177] Santo M. Weitsman R. Sivan A. (2013). The role of the copper-binding enzyme, laccase, in the biodegradation of polyethylene by the actinomycete *Rhodococcus ruber*. *Int. Biodeterior. Biodegrad.* 84 204–210. 10.1016/j.ibiod.2012.03.001

[B178] Schlemmer D. Sales M. J. A. Resck I. S. (2009). Degradation of different polystyrene/thermoplastic starch blends buried in soil. *Carb. Polym.* 75 58–62. 10.1016/j.carbpol.2008.06.010

[B179] Sekhar V. C. Nampoothiri K. M. Mohan A. J. Nair N. R. Bhaskar T. Pandey A. (2016). Microbial degradation of high impact polystyrene (HIPS), an e-plastic with decabromodiphenyl oxide and antimony trioxide. *J. Hazard. Mater.* 318 347–354. 10.1016/j.jhazmat.2016.07.008 27434738

[B180] Sen S. K. Raut S. (2016). Microbial degradation of low density polyethylene (LDPE): a review. *J. Environ. Chem. Eng.* 3 462–473. 10.1016/j.jece.2015.01.003

[B181] Seppala J. Linko Y. Y. Su T. (1991). Photo and biodegradation of high volume thermoplastics, Acta polytechnica scandinavica. *J. Chem. Technol. Metall.* 198:33.

[B182] Shah A. A. Hasan F. Hameed A. Ahmed S. (2008). Biological degradation of plastics: a comprehensive review. *Biotechnol. Adv.* 26 246–265. 10.1016/j.biotechadv.2007.12.005 18337047

[B183] Shah M. M. Barr D. P. Chung N. Aust S. D. (1992). Use of white rot fungi in the degradation of environmental chemicals. *Toxicol. Lett.* 64 493–501. 10.1016/0378-4274(92)90224-8 1281938

[B184] Shang J. Chai M. Zhu Y. (2003). Photocatalytic degradation of polystyrene plastic under fluorescent light. *Environ. Sci. Technol.* 37 4494–4499. 10.1021/es0209464 14572106

[B185] Sharma S. Chatterjee S. (2017). Microplastic pollution, a threat to marine ecosystem and human health: a short review. *Environ. Sci. Pollut. Res.* 24 21530–21547. 10.1007/s11356-017-9910-8 28815367

[B186] Shimpi N. Mishra S. Kadam M. (2012). Biodegradation of polystyrene (PS)-poly(lactic acid) (PLA) nanocomposites using *Pseudomonas aeruginosa*. *Macromol. Res.* 20 181–187. 10.1007/s13233-012-0026-1

[B187] Sielicki M. Focht D. D. Martin J. P. (1978). Microbial degradation of (C14C) polystyrene and 1,3-diphenylbutane. *Can. J. Microbiol.* 24 798–803. 10.1139/m78-134 98222

[B188] Silva C. Da S. Silva N. Matama T. Araujo R. Martins M. (2011). Engineered *Thermobifida fusca* cutinase with increased activity on polyester substrates. *Biotechnol. J.* 6 1230–1239. 10.1002/biot.201000391 21751386

[B189] Song Y. Qiu R. Hu J. Li X. Zhang X. Chen Y. (2020). Biodegradation and disintegration of expanded polystyrene by land snails *Achatina fulica*. *Sci. Total Environ.* 746:141289. 10.1016/j.scitotenv.2020.141289 32745868

[B190] Stern R. V. Howard G. T. (2000). The polyester polyurethanase gene (*pueA*) from *Pseudomonas chlororaphis* encodes a lipase. *FEMS Microbiol. Lett.* 185 163–168. 10.1111/j.1574-6968.2000.tb09056.x 10754242

[B191] Stinson S. (1987). Discoverers of polypropylene share prize. *Chem. Eng. News* 65:30 10.1021/cen-v065n010.p030

[B192] Sudhakar M. Doble M. Sriyutha Murthy P. Venkatesan R. (2008). Marine microbe-mediated biodegradation of low- and high-density polyethylenes. *Int. Biodeterior. Biodegrad.* 61 203–213. 10.1016/j.ibiod.2007.07.011

[B193] Syranidou E. Karkanorachaki K. Amorotti F. Repouskou E. Kroll K. Kolvenbach B. (2017). Development of tailored indigenous marine consortia for the degradation of naturally weathered polyethylene films. *PLoS One* 12:e0183984. 10.1371/journal.pone.0183984 28841722 PMC5571942

[B194] Takei D. Washio K. Morikawa M. (2008). Identification of alkane hydroxylase genes in *Rhodococcus* sp. strain TMP2 that degrades a branched alkane. *Biotechnol. Lett.* 30 1447–1452. 10.1007/s10529-008-9710-9 18414802

[B195] Tokiwa Y. Calabia B. P. (2007). Biodegradability and biodegradation of polyesters. *J. Polym. Environ.* 15 259–267. 10.1007/s10924-007-0066-3

[B196] Tokiwa Y. Calabia B. P. Ugwu C. U. Aiba S. (2009). Biodegradability of plastics. *Int. J. Mol. Sci.* 10 3722–3742. 10.3390/ijms10093722 19865515 PMC2769161

[B197] Torikai A. Hasegawa H. (1999). Accelerated photodegradation of poly (vinyl chloride). *Polym. Degrad. Stab.* 63 441–445. 10.1016/S0141-3910(98)00125-6

[B198] Usha R. Sangeetha T. Palaniswamy M. (2011). Screening of polyethylene degrading microorganisms from garbage soil. *Libyan Agric. Res. Center J. Int.* 2 200–204.

[B199] Vega R. E. Main T. Howard G. T. (1999). Cloning and expression in *Escherichia coli* of apolyurethane-degrading enzyme from *Pseudomonas fluorescens*. *Int. Biodeterior. Biodegrad.* 43 49–55. 10.1016/S0964-8305(98)00068-7

[B200] Venkatachalam S. Nayak S. G. Labde J. V. Gharal P. R. Rao K. Kelkar A. K. (2012). “Degradation and Recyclability of poly (ethylene terephthalate),” in Polyester, ed. Saleh H. E.-D. London: InTech 10.5772/48612

[B201] Verce M. F. Ulrich R. L. Freedman D. L. (2000). Characterization of an isolate that uses vinyl chloride as a growth substrate under aerobic conditions. *Appl. Environ. Microbiol.* 66 3535–3542. 10.1128/AEM.66.8.3535-3542.2000 10919818 PMC92182

[B202] Vertommen M. A. M. E. Nierstrasz V. A. van der Veer M. Warmoeskerken M. M. C. G. (2005). Enzymatic surface modification of poly(ethylene terephthalate). *J. Biotechnol.* 120 376–386. 10.1016/j.jbiotec.2005.06.015 16115695

[B203] Wallace P. W. Haernvall K. Ribitsch D. Zitzenbacher S. Schittmayer M. Steinkellner G. (2017). PpEst is a novel PBAT degrading polyesterase identified by proteomic screening of *Pseudomonas pseudoalcaligenes*. *Appl. Microbiol. Biotechnol.* 101 2291–2303. 10.1007/s00253-016-7992-8 27872998 PMC5320007

[B204] Ward P. G. Goff M. Donner M. Kaminsky W. O’Connor K. E. (2006). A two-step chemo-biotechnological conversion of polystyrene to a biodegradable thermoplastic. *Environ. Sci. Technol.* 40 2433–2437. 10.1021/es0517668 16649270

[B205] Watanabe M. Kawai F. Shibata M. Yokoyama S. Sudate Y. (2003). Computational method for analysis of polyethylene biodegradation. *J. Comput. Appl. Math.* 161 133–144. 10.1016/S0377-0427(03)00551-X

[B206] Webb H. K. Arnott J. Crawford R. J. Ivanova E. P. (2013). Plastic degradation and its environmental implications with special reference to poly(ethylene terephthalate). *Polymers* 5 1–18. 10.3390/polym5010001

[B207] Wehrmann M. Billard P. Martin-Meriadec A. Zegeye A. Klebensberger J. (2017). Functional role of lanthanides in enzymatic activity and transcriptional regulation of pyrroloquinoline quinone-dependent alcohol dehydrogenases in *Pseudomonas putida* KT2440. *mBio* 8:e00570-17. 10.1128/mBio.00570-17 28655819 PMC5487730

[B208] Wei R. Oeser T. Barth M. Weigl N. Lübs A. Schulz-Siegmund M. (2014a). Turbidimetric analysis of the enzymatic hydrolysis of polyethylene terephthalate nanoparticles. *J. Mol. Catal. BEnzym.* 103 72–78. 10.1016/j.molcatb.2013.08.010

[B209] Wei R. Oeser T. Then J. Kühn N. Barth M. Zimmermann W. (2014b). Functional characterization and structural modeling of synthetic polyester-degrading hydrolases form *Thermomonospora curvata*. *AMB Express* 4:44. 10.1186/s13568-014-0044-9 25405080 PMC4231364

[B210] Wei R. Zimmermann W. (2017). Microbial enzymes for the recycling of recalcitrant petroleum-based plastics: how far are we? *Microb. Biotechnol.* 10 1308. 10.1111/1751-7915.12710 28371373 PMC5658625

[B211] Weiland M. Daro A. David C. (1995). Biodegradation of thermally oxidised polyethylene. *Polym. Degrad. Stab.* 48 275–289. 10.1016/0141-3910(95)00040-S

[B212] Welzel K. Müller R. J. Deckwer W. D. (2002). Enzymatischer Abbau von polyester-nanopartikeln. *Chem. Ingenieur Technik* 74 1496–1500. 10.1002/1522-2640(20021015)74:10<1496::AID-CITE1496>3.0.CO;2-P

[B213] Wilkes R. A. Aristilde L. (2017). Degradation and metabolism of synthetic plastics and associated products by *Pseudomonas* sp.: capabilities and challenges. *J. Appl. Microbiol.* 123 582–593. 10.1111/jam.13472 28419654

[B214] Wool R. P. Raghavan D. Wagner G. C. Billieux S. (2000). Biodegradation dynamics of polymer-starch composites. *J. Appl. Polym. Sci.* 77 1643–1657. 10.1002/1097-4628(20000822)77:8<1643::AID-APP1>3.0.CO;2-8

[B215] Xu J. Cui Z. Nie K. Cao H. Jiang M. Xu H. (2019). A quantum mechanism study of the C-C bond cleavage to predict the bio-catalytic polyethylene degradation. *Front. Microbiol.* 10:489. 10.3389/fmicb.2019.00489 30915061 PMC6422906

[B216] Yang J. Yang Y. Wu W. M. Zhao J. Jiang L. (2014). Evidence of polyethylene biodegradation by bacterial strains from the guts of plastic-eating waxworms. *Environ. Sci. Technol.* 48 13776–13784. 10.1021/es504038a 25384056

[B217] Yang L. Gao J. Liu Y. Zhuang G. Peng X. Wu W.-M. (2021). Biodegradation of expanded polystyrene and low-density polyethylene foams in larvae of *Tenebrio molitor* Linnaeus (Coleoptera: Tenebrionidae): broad versus limited extent depolymerization and microbe-dependence versus independence. *Chemosphere* 262:127818. 10.1016/j.chemosphere.2020.127818 32771707

[B218] Yang S.-S. Brandon A. M. Andrew Flanagan J. C. Yang J. Ning D. Cai S.-Y. Y. (2018a). Biodegradation of polystyrene wastes in yellow mealworms (larvae of *Tenebrio molitor* Linnaeus): factors affecting biodegradation rates and the ability of polystyrene-fed larvae to complete their life cycle. *Chemosphere* 191 979–989. 10.1016/j.chemosphere.2017.10.117 29145143

[B219] Yang S.-S. Wu W. M. Brandon A. M. Fan H. Q. Receveur J. P. Li Y. (2018b). Ubiquity of polystyrene digestion and biodegradation within yellow mealworms, larvae of *Tenebrio molitor* Linnaeus (Coleoptera: Tenebrionidae). *Chemosphere* 212 262–271. 30145418 10.1016/j.chemosphere.2018.08.078

[B220] Yang Y. Chen J. Wu W.-M. Zhao J. Yang J. (2015). Complete genome sequence of *Bacillus* sp.YP1, a polyethylene-degrading bacterium from waxworm’s gut. *J. Biotechnol.* 200 77–78. 10.1016/j.jbiotec.2015.02.034 25795022

[B221] Yang Y. Yang J. Wu W. M. Zhao J. Song Y. Gao L. (2015a). Biodegradation and mineralization of polystyrene by plastic-eating mealworms. 1. Chemical and physical characterization and isotopic tests. *Environ. Sci. Technol.* 49:12080. 10.1021/acs.est.5b02661 26390034

[B222] Yang Y. Yang J. Wu W. M. Zhao J. Song Y. Gao L. (2015b). Biodegradation and mineralization of polystyrene by plastic-eating mealworms: Part 2. Role of gut microorganisms. *Environ. Sci. Technol.* 49 12087–12093. 10.1021/acs.est.5b02663 26390390

[B223] Yin C.-F. Xu Y. Zhou N.-Y. (2020). Biodegradation of polyethylene mulching films by a co-culture of *Acinetobacter* sp. strain NyZ450 and *Bacillus* sp. strain NyZ451 isolated from *Tenebrio molitor* larvae. *Int. Biodeterior. Biodegrad.* 155:105089 10.1016/j.ibiod.2020.105089

[B224] Yoon M. G. Jeon J. H. Kim M. N. (2012). Biodegradation of polyethylene by a soil bacterium and AlkB cloned recombinant cell. *J. Bioremed. Biodegrad.* 3:145.

[B225] Yoshida S. Hiraga K. Takehara T. Oda K. (2016). A bacterium that degrades and assimilates poly(ethylene terephthalate). *Science* 351 1196–1199. 10.1126/science.aad6359 26965627

[B226] Yue H. Zhao Y. Ma X. Gong J. (2012). Ethylene glycol. Properties, synthesis, and applications. *Chem. Soc. Rev.* 41 4218–4244. 10.1039/c2cs15359a 22488259

[B227] Zhang Y. Chen S. Xu M. Cavaco-Paulo A. Wu J. Chen J. (2010). Characterization of *Thermobifida fusca* cutinase-carbohydrate-binding module fusion proteins and their potential application in bioscouring. *Appl. Environ. Microbiol.* 76 6870–6876. 10.1128/AEM.00896-10 20729325 PMC2953015

[B228] Zheng Y. Yanful E. K. (2005). A review of plastic waste degradation. *Crit. Rev. Biotechnol.* 25 243–250. 16419620 10.1080/07388550500346359

[B229] Zheng Y. Yanful E. K. Bassi A. S. (2005). A review of plastic waste biodegradation. *Crit. Rev. Biotechnol.* 25 243–250. 10.1080/07388550500346359 16419620

[B230] Zimmermann W. Billig S. (2011). Enzymes for the biofunctionalization of poly(ethylene terephthalate). *Adv. Biochem. Engin/Biotechnol.* 125 97–120. 10.1007/10_2010_87 21076908

[B231] Zuchowska D. Hlavata D. Steller R. Adamiak W. Meissner W. (1999). Physical structure of polyolefin-starch blends after ageing. *Polym. Degrad. Stab.* 64 339–347. 10.1016/S0141-3910(98)00212-2

[B232] Zuchowska D. Steller R. Meissner W. (1998). Structure and properties of degradable polyolefin-starch blends. *Polym. Degrad. Stab.* 60 471–480. 10.1016/S0141-3910(97)00110-9

[B233] Zumstein M. T. Rechsteiner D. Roduner N. Perz V. Ribitsch D. Guebitz G. M. (2017). Enzymatic hydrolysis of polyester thin films at the nanoscale: effects of polyester structure and enzyme active-site accessibility. *Environ. Sci. Technol.* 51 7476–7485. 10.1021/acs.est.7b01330 28538100

